# Molecular genetics of syndromic and non‐syndromic forms of parathyroid carcinoma

**DOI:** 10.1002/humu.23337

**Published:** 2017-09-25

**Authors:** Luís Cardoso, Mark Stevenson, Rajesh V. Thakker

**Affiliations:** ^1^ Department of Endocrinology Diabetes and Metabolism Centro Hospitalar e Universitário de Coimbra Praceta Prof Mota Pinto Coimbra Portugal; ^2^ Radcliffe Department of Medicine Academic Endocrine Unit Oxford Centre for Diabetes Endocrinology and Metabolism University of Oxford Oxford United Kingdom

**Keywords:** *CDC73*, familial isolated primary hyperparathyroidism, genetic syndromes, hyperparathyroidism‐jaw tumor syndrome, multiple endocrine neoplasia type 1

## Abstract

Parathyroid carcinoma (PC) may occur as part of a complex hereditary syndrome or an isolated (i.e., non‐syndromic) non‐hereditary (i.e., sporadic) endocrinopathy. Studies of hereditary and syndromic forms of PC, which include the hyperparathyroidism‐jaw tumor syndrome (HPT‐JT), multiple endocrine neoplasia types 1 and 2 (MEN1 and MEN2), and familial isolated primary hyperparathyroidism (FIHP), have revealed some genetic mechanisms underlying PC. Thus, cell division cycle 73 (*CDC73*) germline mutations cause HPT‐JT, and *CDC73* mutations occur in 70% of sporadic PC, but in only ∼2% of parathyroid adenomas. Moreover, *CDC73* germline mutations occur in 20%–40% of patients with sporadic PC and may reveal unrecognized HPT‐JT. This indicates that *CDC73* mutations are major driver mutations in the etiology of PCs. However, there is no genotype–phenotype correlation and some *CDC73* mutations (e.g., c.679_680insAG) have been reported in patients with sporadic PC, HPT‐JT, or FIHP. Other genes involved in sporadic PC include germline *MEN1* and rearranged during transfection (*RET*) mutations and somatic alterations of the retinoblastoma 1 (*RB1*) and tumor protein P53 (*TP53*) genes, as well as epigenetic modifications including DNA methylation and histone modifications, and microRNA misregulation. This review summarizes the genetics and epigenetics of the familial syndromic and non‐syndromic (sporadic) forms of PC.

## 1 INTRODUCTION

Parathyroid carcinoma (PC) is a rare endocrine malignancy accounting for 0.005% of all cancers and <1% of primary hyperparathyroidism (pHPT) (Hundahl, Fleming, Fremgen, & Menck, 1999; Ruda, Hollenbeak, & Stack, 2005). Data from the Surveillance, Epidemiology, and End Results cancer registry showed a 60% increase in PC incidence from 1988 to 2003, which, in part, may be due to increased screening of serum calcium and an increased number of patients undergoing surgery for asymptomatic pHPT (Lee, Jarosek, Virnig, Evasovich, & Tuttle, 2007). PC was first reported in 1909 by the Swiss surgeon Fritz de Quervain in a 68‐year‐old man who presented with a large neck mass and died from local recurrence and pulmonary metastasis (Quervain, 1909). Most PCs secrete parathyroid hormone (PTH) resulting in hypercalcemia, however, approximately 40 PC cases have been reported in which there was no increase in PTH production and morbidity resulted from tumor invasion and spread (Wang et al., 2015). PC, parathyroid adenoma (PA), and atypical parathyroid adenoma (APA) cannot be reliably distinguished on the basis of plasma concentrations of calcium and PTH in individual patients, although plasma calcium and PTH concentrations are often higher in patients with PC than patients with PA. Thus, the diagnosis of PC relies on histological criteria, which require demonstration of either capsular invasion with growth into adjacent tissues, vascular and/or perineural tumor invasion, and/or metastasis (Bondeson, et al., 2004). Moreover, the presence of four or more associated features of malignancy that include: capsular invasion without extension to surrounding soft tissue; mitosis >5/10 high power fields; broad intratumoral fibrous bands; coagulative tumor necrosis; diffuse sheet‐like monotonous small cells with high nucleus:cytoplasmic ratio; diffuse cellular atypia; and presence of macronuclei in many tumor cells, qualifies for a diagnosis of PC, whereas the presence of only one to three of these features, qualifies for a diagnosis of APA, which is considered to have features of carcinomas that lack unequivocal evidence for invasive growth (Bondeson, et al., 2004; Chan, 2013; DeLellis, 2011; Kumari, Chaudhary, Pradhan, Agarwal, & Krishnani, 2016). Indeed, using such clinicopathological criteria only ∼15%–35% of the prospectively diagnosed PC cases will continue to behave in a malignant manner, whereas ≤50% of PCs will have an initial diagnosis of benign disease (Gill, 2014; Marsh, Hahn, Howell, & Gill, 2007). Prospective diagnosis of PC is important, as cure can only be achieved following complete surgical resection. Therefore, a broader understanding of the molecular and hereditary basis of PC would provide insight to improve pre‐ and post‐surgical diagnosis and staging.

The aim of this review is to summarize the current knowledge of the molecular and hereditary basis of PC. A PubMed and EMBASE literature search was undertaken on July 1, 2016 using the search term: “parathyroid carcinoma”, its free and controlled vocabulary EMTREE and MeSH synonyms, cross‐referenced with “genetics,” “epigenetics,” “mutations,” “*CDC73*,” “hyperparathyroidism‐jaw tumor,” “familial isolated hyperparathyroidism,” “MEN1,” “MEN2,” and its free and controlled vocabulary EMTREE and MeSH synonyms. There were no restrictions on language, publication type, or date. Additionally, reference lists from all major reviews were examined for citations that did not appear in the PubMed or EMBASE search.

## 2 CLINICAL FEATURES OF PARATHYROID CARCINOMA

**Figure 1:**
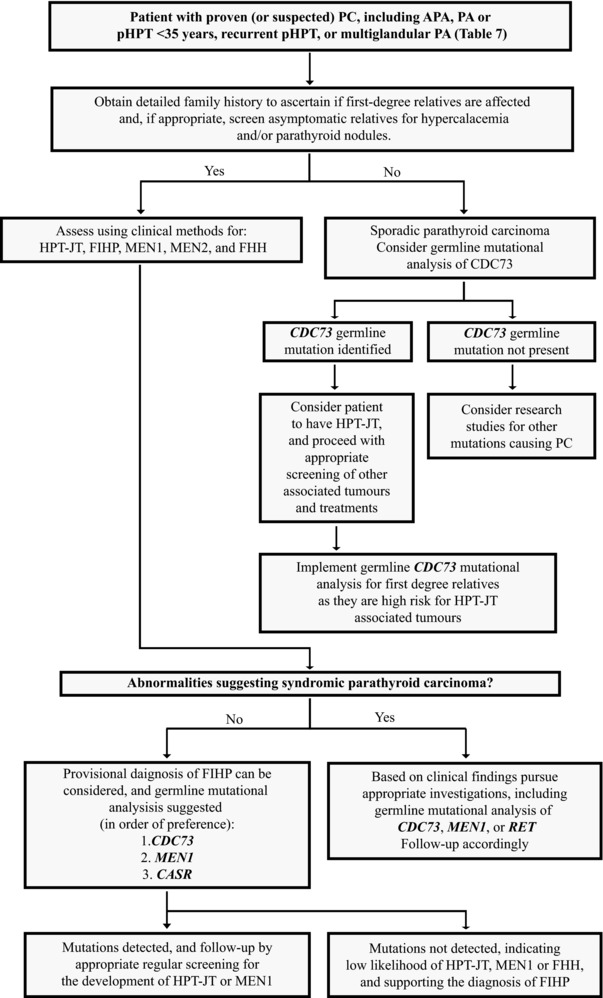
A genetic testing approach to patients with parathyroid carcinoma. PC, parathyroid carcinoma; APA, atypical parathyroid adenoma; PA, parathyroid adenoma; pHPT, primary hyperparathyroidism; HPT‐JT, hyperparathyroidism‐jaw tumor; FIHP, familial isolated primary hyperparathyroidism; MEN1, multiple endocrine neoplasia type 1; MEN2, multiple endocrine neoplasia type 2; FHH, familial hypocalciuric hypercalcemia

PC may occur as part of a complex syndrome and hereditary disorder, or as a non‐hereditary (i.e., sporadic) and isolated (i.e., non‐syndromic) endocrinopathy (Table 1 and Figure 1). PC most commonly occurs as a sporadic non‐syndromic disorder. The hereditary syndromes associated with PC include the hyperparathyroidism‐jaw tumor (HPT‐JT) syndrome, the multiple endocrine neoplasia (MEN) type 1 (MEN1) and type 2 (MEN2) syndromes, and potentially the non‐syndromic familial isolated primary hyperparathyroidism (FIHP), which may be clinically difficult to distinguish from the MEN1 and HPT‐JT syndromes (Figure 1).

**Table 1:** Syndromic and hereditary forms of parathyroid carcinoma

Condition^a^	Syndromic or isolated	Gene affected	Chromosomal location^b^	Protein function^c^	Inheritance^d^	pHPT features^e^	Associated conditions^f^
HPT‐JT	Syndromic	*CDC73*	1q31.2	TS	AD	PA (cystic)/PC	Jaw, renal, and uterine tumors
FIHP	Isolated	*CDC73* *MEN1*	1q31.2 11q13	TS TS	AD^g^	PA/PC Hyperplasia/PA/PC	
MEN1	Syndromic	*MEN1*	11q13	TS	AD	Hyperplasia/PA/PC	Enteropancreatic tumors (75%), pituitary (50%), and adrenal hyperplasia (13%) or tumors (13%)
MEN2	Syndromic	*RET*	10q11.21	Onco	AD	Hyperplasia/PA/PC	MTC (66%) and pheochromocytoma (33%)

a HPT‐JT, hyperparathyroidism‐jaw tumor; FIHP, familial isolated primary hyperparathyroidism; MEN1, multiple endocrine neoplasia type 1; MEN2, multiple endocrine neoplasia type 2.

b Cytogenetic band according to HUGO Gene Nomenclature Committee.

c TS, tumor suppressor; Onco, proto‐oncogene.

d AD, autosomal dominant.

e pHPT, primary hyperparathyroidism; PA, parathyroid adenoma; PC, parathyroid carcinoma.

f MTC, medullary thyroid cancer.

g Some families may show autosomal recessive inheritance.

The clinical findings of PC are generally non‐specific and the diagnosis of PC is rarely made before surgery and histological examination of the tumor. Thus, distinguishing between benign and malignant disease is a challenge in the management of patients with pHPT. The most frequent symptoms of PC are those associated with hypercalcemia and are: fatigue, weakness, weight loss, anorexia, nausea, vomiting, abdominal pain, polyuria, and polydipsia. Other clinical features may include bone pain, fractures, anemia, nephrolithiasis, pancreatitis, and peptic ulcer disease (Busaidy et al., 2004; Chen et al., 2003; Hakaim & Esselstyn, 1993; Schantz & Castleman, 1973; Wynne, van Heerden, Carney, & Fitzpatrick, 1992). Renal and bone involvement is frequent and may coexist in >50% of PC patients (Wynne et al., 1992). Osteoporosis, *osteitis fibrosa cystica*, subperiosteal bone resorption, or salt‐and‐pepper skull lesions have been reported to occur in ∼40%–90% of PC patients, whereas bone disease occurs in <10% of patients with benign pHPT (Schantz & Castleman, 1973; Shane & Bilezikian, 1982; Wang & Gaz, 1985; Silverberg et al., 1990; Wynne et al., 1992). Renal involvement has been reported to occur in ∼30%–85% of PC patients with nephrocalcinosis occurring in ∼15%–55%, nephrolithiasis in ∼55%–70%, and renal insufficiency in ∼55%–85% of patients (Holmes, Morton, & Ketcham, 1969; Schantz & Castleman, 1973; Shane & Bilezikian, 1982; Wynne et al., 1992). Conversely, renal involvement in benign pHPT is considerably lower, affecting ∼20% of patients (Silverberg et al., 1990).

A palpable tumor is found in ∼50% of PC patients, whereas it is rarely identifiable in patients with benign pHPT (Holmes et al., 1969; Wynne et al., 1992). More than 90% of PC cases involve functioning tumors with plasma PTH concentrations 3–10 times higher than normal upper limit, whereas plasma PTH concentrations 2–3 times higher are typically found in benign pHPT (Holmes et al., 1969; Wynne et al., 1992). Recently, a population‐based study reported a positive predictive value of >80% for PTH levels ≥10 times higher than the upper normal limit (Schaapveld et al., 2011). Most PC patients have severe hypercalcemia at presentation (calcium >14 mg/dl, i.e.,  >3.50 mmol/l), whereas in benign pHPT calcium levels are generally 1–2 mg/dl (i.e., 0.25–0.50 mmol/l) above normal (Wang & Gaz, 1985; Wynne et al., 1992; Chen et al., 2003). Plasma alkaline phosphatase activity is more commonly elevated in patients with PC than benign pHPT as a result of bone involvement (Silverberg et al., 1990; Chen et al., 2003). However, there is considerable overlap of these elevations of plasma calcium and PTH concentrations and alkaline phosphatase activity in patients with PC and PA, thereby making it difficult to rely upon them for establishing an unequivocal diagnosis of PC. However, PC patients have been reported to have elevated levels of urinary human chorionic gonadotropin subunits, particularly the hyperglycosylated isoforms, which are associated with an increased risk of hip fracture and death, and this difference from patients with benign pHPT requires further study (Rubin, Bilezikian, Birken, & Silverberg, 2008).

## 3 SYNDROMIC AND HEREDITARY FORMS OF PARATHYROID CARCINOMA

The syndromic and hereditary forms of PC are associated with germline mutations of the cell division cycle 73 (*CDC73*) gene, also referred to as the hyperparathyroidism type 2 (*HRPT2*) gene, MEN type 1 (*MEN1*), and rearranged during transfection (*RET*) genes (Table 1). The *RET* mutations, which are activating, are dominant at the cellular level, and only one copy of the mutated gene is required for tumor development. However, for the *MEN1* and *CDC73* mutations, which are inactivating and recessive at the cellular level, two mutations are required for a tumor to develop: for the hereditary tumors, these two recessive mutations comprise one germline and one somatic mutation that may involve a chromosomal loss and be detected as loss of heterozygosity (LOH) in the tumor. Such tumors may also occur sporadically, that is, without a family history and without inheritance of the germline mutation, and in these patients, both the recessive mutations will have likely occurred as somatic mutations in the tumor. This genetic model of neoplasia involving two recessive mutations in the development of tumors is known as Knudson's two‐hit hypothesis. The genetic mechanisms involved in the etiology of the MEN1 and HPT‐JT syndromes due to *MEN1* and *CDC73* mutations are consistent with Knudson's two‐hit hypothesis (Knudson, 1971; Thakker, 1993).

### 3.1 Hyperparathyroidism‐jaw tumor (HPT‐JT)

HPT‐JT (MIM# 145001) is a rare syndrome characterized by pHPT, fibro‐osseous lesions (ossifying fibroma) of the mandible and maxilla, and tumors of the kidney and uterus (Jackson, 1958; Bradley et al., 2005b). Parathyroid tumors, of which 15% are carcinomas, are generally the first manifestation, and occur in >90% of HPT‐JT cases (Bradley & Thakker, 2006). pHPT is usually caused by a solitary parathyroid tumor, but multiglandular involvement may affect >15% of cases (Marx, 2000; Bradley & Thakker, 2006; Mehta et al., 2014).

### 3.1.1 CDC73

**Figure 2:**
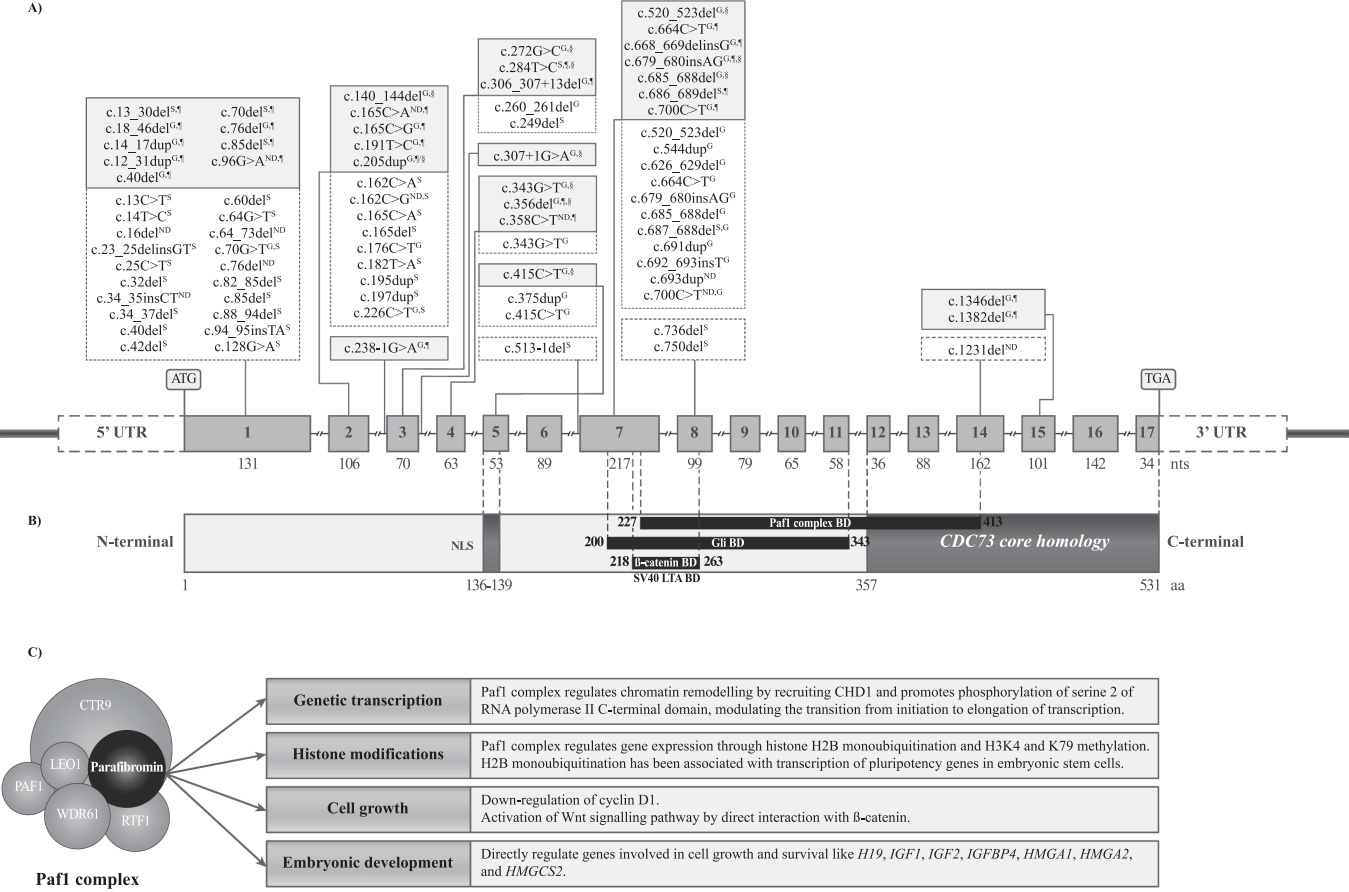
Schematic representation of the genomic organization of the human *CDC73* gene, parafibromin protein, and its functions. (A) Upper panel, schematic representation of genomic structure of cell division cycle 73 (*CDC73*) comprising 17 exons. ATG and TGA represent the initiation and stop codons, respectively. Sites of *CDC73* mutations associated with sporadic and familial parathyroid carcinoma (PC) are shown (^S^ somatic mutation; ^G^ germline mutation; ^ND^ not defined; white, dotted line boxes, *CDC73* mutations associated with sporadic PC; gray, full line boxes, *CDC73* mutations associated with syndromic or hereditary forms of PC, where ^¶^ means hyperparathyroidism‐jaw tumor and ^§^ means familial isolated primary hyperparathyroidism). (B) Middle panel, schematic representation of parafibromin protein structure and known functional domains. *CDC73* encodes a 531‐amino acid protein, whose C‐terminal domain shares 27% homology with the yeast CDC73 (CDC73 core homology domain). The nuclear localization signal (NLS) is encoded by exon 5, the evolutionary conserved polymerase‐associated factor 1 (Paf1) complex‐binding domain (Paf1 complex BD) by exons 7–14, the Gli binding domain (Gli BD) by exons 7–11, and the β‐catenin interaction binding domain (β‐catenin BD) and the SV40 large T antigen binding domain (SV40 LTA BD) by exons 7 and 8. (C) Lower panel, schematic representation of parafibromin functions. Parafibromin is a component of the Paf1 protein complex, which regulates chromatin remodeling and gene expression via histone modification. Parafibromin also regulates cell growth, via cyclin D1 and Wnt signaling, and embryonic development via genes involved in cell growth and survival. *H19*, H19 fetal liver mRNA; *IGF1* and *IGF2*, insulin‐like growth factor 1 and 2; *IGFBP4*, insulin‐like growth factor binding protein 4; *HMGA1* and *HMGA2*, high mobility AT‐hook 1 and 2; *HMGCS2*, 3‐hydroxy‐3‐methylglutaryl‐Coenzyme A synthase 2

HPT‐JT is an autosomal dominant disease due to germline mutations of the *CDC73* gene (Tables 1, 2, 3). *CDC73*, which is comprised of 17 exons (Figure 2A) and is located on chromosome 1q31.2, encodes the protein, parafibromin, which is associated in the polymerase associated factor (Paf1) complex (Figure 2B and C) with the proteins: PAF1; tryptophan‐aspartic acid dipeptide terminating repeat domain 61 (WDR61); and the RNA polymerase‐associated proteins—left open reading frame homolog (LEO1), cyclin three requiring homolog (CTR9), and restores TATA‐binding protein function homolog (RTF1). The Paf1 complex interacts with the RNA polymerase II subunit A (POLR2A), regulating genetic transcription, and with the histone methyltransferase complex, regulating histone modifications (Figure 2C) (Rozenblatt‐Rosen et al., 2005; Yart et al., 2005). Functions attributed to parafibromin include the downregulation of cyclin D1 expression and direct interaction with β‐catenin resulting in the activation of transcription of target genes (Figure 2C) (Woodard et al., 2005; Mosimann, Hausmann, & Basler, 2006; Zhang et al., 2006; Bradley et al., 2007). Parafibromin also has a role in embryonic development regulating genes involved in cell growth and survival (Figure 2C) (Wang et al., 2008).

**Table 2:** Spectrum of diseases associated with *CDC73* mutations

Hyperparathyroidism‐jaw tumor
Diagnosis may be established in individuals with: pHPT and ossifying fibroma(s) of the maxilla and/or mandible, or pHPT and a direct relative with HPT‐JT, or Ossifying fibroma(s) of the maxilla and/or mandible and a direct relative with HPT‐JT

*CDC73*‐related familial isolated primary hyperparathyroidism
Diagnosis may be established in individuals with: pHPT and a *CDC73* germline pathogenic variant, and At least one relative with pHPT, and Absence of ossifying fibromas and exclusion of other causes of familial hyperparathyroidism

*CDC73*‐related parathyroid carcinoma
Diagnosis may be established in individuals with: PC and a *CDC73* germline pathogenic variant Germline mutations often occur simultaneously with somatic mutations. PC is the most severe form of *CDC73* associated diseases and may occur isolated or in the context of HPT‐JT or FIHP

pHPT, primary hyperparathyroidism; HPT‐JT, hyperparathyroidism‐jaw tumor; PC, parathyroid carcinoma; FIHP, familial isolated primary hyperparathyroidism.

**Table 3:** Summary of *CDC73* mutations associated with hyperparathyroidism‐jaw tumor

**Mutation** a	**Exon/intron**	**Codon** b	**Predicted effect** c	**Type** d	**Original designation**	**References**
c.‐16_8del	Exon 1		p.Met?	G	c.‐16:8del; p.Met1?	Bellido et al. (2016)
c.3G > A	Exon 1	1	p.Met?	G	3G→A	Carpten et al. (2002)^r^
c.13_30del	Exon 1	5	p.Leu5_Gln10del	S^p^ ^,1^	13_30delCTTAGCGTCCTGCGACAG	Moon et al. (2005)
c.18_46del	Exon 1	6	p.Ser6ArgfsX50	G^p^	c.18_48del31	Parfitt, Harris, Wright, and Kalamchi (2015)
c.14_17dup	Exon 1	7	p.Val7X	G^p^	c.14_17dupTTAG	Khadilkar et al. (2015)^r^^,^ e
c.[24del;20T > C]^q^	Exon 1	7	p.Val7AlafsX14	G	nt20AGGACG→GGGAG	Aldred et al. (2006)
c.22del	Exon 1	8	p.Leu8CysfsX13	G	c.22delC	Carlson & Smith (2008)
c.25C > T	Exon 1	9	p.Arg9X	G	25C→T	Carpten et al. (2002)^r^
c.25C > T	Exon 1	9	p.Arg9X	G	c.25 > T	Newey et al. (2010)^f^
c.25C > T	Exon 1	9	p.Arg9X	ND	R9X	Schmidt, Bradrick, and Gabali (2009)
c.25C > T	Exon 1	9	p.Arg9X	ND	p.Arg9Stop (R9X)	Mathews, Winchester, Alsaygh, Bartlett, and Luttrell (2016)
c.30del	Exon 1	10	p.Gln10HisfsX11	G	30delG	Carpten et al. (2002)^r^
c.12_31dup	Exon 1	11	p.Tyr11CysfsX17	G^p^	41 bp duplication/insertion	Carpten et al. (2002)^r^
c.35_41del	Exon 1	12	p.Asn12ArgfsX7	G	34delAACATCC	Carpten et al. (2002)^r^
c.40C > T	Exon 1	14	p.Gln14X	G	c.40C > T	Khadilkar et al. (2015)
c.40del	Exon 1	14	p.Gln14ArgfsX7	G^p^	39delC	Carpten et al. (2002)^r^
c.40del	Exon 1	14	p.Gln14ArgfsX7	G	39delC	Mizusawa et al. (2006)
c.40del	Exon 1	14	p.Gln14ArgfsX7	G	39delC	Yamashita, Akiyama, Mizusawa, Yoshimoto, and Goto (2007)
c.70del	Exon 1	24	p.Glu24LysfsX2	S^p^	c.70delG	Sriphrapradang et al. (2014)
c.76del	Exon 1	26	p.Ile26SerfsX11	G	c.76delA	Howell et al. (2003)^g^^,^ h
c.76del	Exon 1	26	p.Ile26SerfsX11	G^p^ ^,2^	c.76delA	Howell et al. (2003)^g^^,^ h
c.76del	Exon 1	26	p.Ile26SerfsX11	G^p^	c.76delA	Frank‐Raue et al. (2011)
c.85del	Exon 1	29	p.Glu29SerfsX8	S^p^ ^,1^	85delG	Moon et al. (2005)
c.85del	Exon 1	29	p.Glu29SerfsX8	G	85del	Rekik et al. (2010)
c.85G > T	Exon 1	29	p.Glu29X	G	c.93G > T exon 1	Bricaire et al. (2013)
c.85G > T	Exon 1	29	p.Glu29X	G	c.85G > T	Abdulla, O'Leary, Isorena, Diaz, and Yeh, (2013)
c.96G > A	Exon 1	32	p.Trp32X	G	c.96G > A	Sarquis et al. (2008)^h^^,^ i
c.96G > A	Exon 1	32	p.Trp32X	ND^p^	c.96G > A	Kutcher et al. (2013)
c.131+1G > A	Intron 1		splice [d]^o^	G	c.131+1G > A	Newey et al. (2010)^f^
c.140_144del	Exon 2	47	p.Lys47ArgfsX17	G^3^	c.136_144 del5	Iacobone et al. (2009)^h^^,^ j
c.165C > G	Exon 2	55	p.Tyr55X	G^p^	165C‐G	Carpten et al. (2002)^r^
c.165C > A	Exon 2	55	p.Tyr55X	ND^p^	c.165C > A	Veiguela, Isidro, Jorge, and Ruano (2010)
c.179T > A	Exon 2	60	p.Ile60Asn	S^3^	c.179T > A	Masi et al. (2014)
c.188T > C	Exon 2	63	p.Leu63Pro	G	c.188T > C	Newey et al. (2010)^f^
c.188T > C	Exon 2	63	p.Leu63Pro	G^4^	c.188T > C	Iacobone et al. (2009)^h^^,^ j
c.191T > C	Exon 2	64	p.Leu64Pro	G^p^	L64P	Hahn et al. (2010)^g^^,^ h ^,^ k
c.205dup	Exon 2	69	p.Leu69ProfsX13	G^p^	c.205dupC	Pichardo‐Lowden, Manni, Saunders and Baker (2011)^l^
c.226C > T	Exon 2	76	p.Arg76X	G	c.226C > T	Newey et al. (2010)^f^
c.238‐1G > A	Intron 2		splice [a]^o^	G^p^ ^,1^	IVS2‐1G > A	Moon et al. (2005)
c.284T > C	Exon 3	95	p.Leu95Pro	G	L95P	Panicker, Zhang, Dagur, Gastinger and Simonds, (2010)
c.284T > C	Exon 3	95	p.Leu95Pro	S^p^ ^,5^	c.284T > C	Yu et al. (2015)^h^
c.306_307+13del	Exon 3	103	p.Ser103AsnfsX5	G^p^	*306delGTgtgagtacttttt	Carpten et al. (2002)^r^
c.307+5G > T	Intron 3		splice [vus]	G	c.307+5G > T	Frank‐Raue et al. (2011)
c.356del	Exon 4	119	p.Gln119ArgfsX14	G^p^ ^,5^	356delA	Carpten et al. (2002)^r^^,^ h
c.358C > T	Exon 4	120	p.Arg120X	ND^p^	c.358C > T	Mele, Rolighed, Jespersen, Rejnmark and Christiansen (2016)
c.374_375dup	Exon 5	126	p.Arg126AsnfsX8	S^4^	c.375_376insAA	Masi et al. (2008)^h^
c.406A > T	Exon 5	136	p.Lys136X	G	406A→T	Carpten et al. (2002)^r^
c.433_442delinsAGA	Exon 5	145	p.Cys145ArgfsX55	G	c.433_442delinsAGA	Iacobone et al. (2009)
c.424‐5T > C	Intron 5		splice [vus]	G	c.424‐5T > C	Frank‐Raue et al. (2011)
c.639del	Exon 7	213	p.Phe213LeufsX6	G	636delT	Carpten et al. (2002)^r^
c.664C > T	Exon 7	222	p.Arg222X	G^p^	c.664C > T	Wang et al. (2012)^r^
c.668_669delinsG	Exon 7	223	p.Asp223GlyfsX34	G^p^	669delAT/insG	Bradley et al. (2005b)^r^^,^ h
c.679_680insAG	Exon 7	227	p.Arg227LysfsX31	G	679insAG	Bradley et al. (2005b)^r^^,^ h
c.679_680insAG	Exon 7	227	p.Arg227LysfsX31	G^p^	679insAG	Carpten et al. (2002)^r^^,^ h
c.686_689del	Exon 7	229	p.Arg229AsnfsX27	S^p^ ^,2^	c.686delGAGT	Howell et al. (2003)^g^^,^ h ^,^ m
c.687_688del	Exon 7	229	p.Arg229SerfsX37	G	c.679delAG	Howell et al. (2003)^g^^,^ h
c.687_688del	Exon 7	229	p.Arg229SerfsX37	G	c.679delAG	Sarquis et al. (2008)
c.687_688del	Exon 7	229	p.Arg229SerfsX37	G	AGCACA^GAGAGagTATGGAGGACA	Teh et al. (2004)^f^^,^ n
c.687_688del	Exon 7	229	p.Arg229SerfsX37	G	c.687_688del	Newey et al. (2010)^f^
c.700C > T	Exon 7	234	p.Arg234X	G	700C→T	Bradley et al. (2006)
c.700C > T	Exon 7	234	p.Arg234X	G^p^	R234X	Raue, Haag and Frank‐Raue (2007)
c.700C > T	Exon 7	234	p.Arg234X	G	c.700C > T	Newey et al. (2010)^f^
c.745dup	Exon 8	249	p.Ile249AsnfsX18	G	c.745dupA	Newey et al. (2010)^f^
c.766_767del	Exon 8	256	p.Val256LysfsX10	G	255delTG/256delGT	Cavaco et al. (2004)
c.1126_1127insTT	Exon 13	276	p.Asn376IlefsX10	G	1126InsTT	Pimenta et al. (2006)
c.1135G > A	Exon 13	379	p.Asp379Asn	G	1135 G → A	Bradley et al. (2006)
c.1239del	Exon 14	413	p.Gln413HisfsX15	G	1238delA	Carpten et al. (2002)^r^
c.1247del	Exon 14	416	p.Gly416AlafsX12	G	c.1247delG	Howell et al. (2009)^f^
c.1346del	Exon 15	449	p.Gly449ValfsX30	G^p^	c.1346delG	Frank‐Raue et al. (2011)
c.1382del	Exon 15	461	p.Leu461CysfsX18	G^p^	c.1379delT	Chiofalo et al. (2014)
c.1432_1433del	Exon 16	478	p.Leu478GlufsX3	G	c.1432_1433delCT	Frank‐Raue et al. (2011)
c.*12C > A	3′‐UTR		Expression [vus]	G	c.*12C > A	Frank‐Raue et al. (2011)
Gross deletion				G	c.307+?_513‐?del exons 4, 5, 6	Kong et al. (2014)
Gross deletion				G	c.307+?_513‐?del exons 4, 5, 6	Bricaire et al. (2013)
Gross deletion				G^p^	1q31,1–1q31,3 del	Bricaire et al. (2013)^r^
Gross deletion				G	Whole gene deletion	Cascon et al. (2011)
Gross deletion				G^p^	Whole gene deletion	Bricaire et al. (2013)^r^

a Mutations are numbered in relation to the cell division cycle 73 (*CDC73*) cDNA reference sequence (GenBank accession number NM_024529.4) whereby nucleotide +1 corresponds to the A of the ATG‐translation initiation codon. All mutations were analyzed using the Leiden Open Variation Database (LOVD) Mutalyzer sequence variant nomenclature checker (https://www.lovd.nl/mutalyzer/) and annotated using the Human Genome Variation Society (HGVS) guidelines (https://www.hgvs.org/).

b Codon numbering starts from initiation codon of *CDC73* mRNA.

c Predicted effect: splice, splice site mutation; [d] donor splice site; [a] acceptor splice site; [vus] variant of unknown significance; ? indicates unlikely translation of protein as initiator met is lost.

d Mutation type: G, germline; S, somatic; ND, not defined. Equal superscript numbers represent germline and/or somatic mutations occurring in the same patient.

e Criteria for diagnosis of PC were not reported, but the patient had persistent disease and clinical suspicion of thoracic metastasis.

f Reported as HPT‐JT, but the authors did not provide details about the presence or absence of jaw tumors.

g Additional clinical details about these kindreds are provided Bradley et al. (2005b).

h Reported as HPT‐JT, but occurrence of jaw tumors, which may not always occur in HPT‐JT patients, was not detected in any family members.

i Reported in other publication as a possible FIHP case, but the frequent recurrence, presence of APA and renal and uterine tumors favors the diagnosis of HPT‐JT (Silveira et al., 2008).

j Initially reported as FIHP by Masi et al. (2008).

k Initially reported as FIHP by Howell et al. (2003).

l It is possible this is a case of HPT‐JT associated with PC since: the patient was diagnosed with three renal cysts, while “a maternal cousin had jaw pain and presumably bone destruction of the jaw, termed a ‘hole in the jaw’.” Furthermore, histological description of the proband's parathyroid gland was consistent with an APA (“…vascular and capsular invasion, but no definitive features of PC were identified”) and disease recurrence on the contra‐lateral side (again with diagnosis of APA) suggests a more malignant behavior.

m Reported as a germline mutation in a later publication, but inconsistency between patients’ gender and age are observed (Sarquis et al., 2008).

n Unclear if this kindred was included in the previous study of Howell et al. (2003).

o For detailed information of the effect of *CDC73* mutation on splicing please consult Hahn, McDonnell, and Marsh (2009).

p Mutations identified in kindreds with case reports of PC.

q Discordant codon/nucleotide change in the original report.

r Criteria for diagnosis of PC not reported.

PC, parathyroid carcinoma; HPT‐JT, hyperparathyroidism‐jaw tumor; FIHP, familial isolated primary hyperparathyroidism; APA, atypical parathyroid adenoma.

About 75% of HPT‐JT patients will have germline *CDC73* mutations within the coding region (Table 3 and Figure 2A and B), and the PCs will usually have LOH of *CDC73* resulting in a loss of parafibromin expression. The ∼25% of HPT‐JT families, who do not harbor *CDC73* mutations or deletions of the coding region or adjacent splice sites, may have abnormalities involving the *CDC73* promoter regions, untranslated regions, uncharacterized alternate transcripts, whole exon or gene deletions that are not readily detected by polymerase chain reaction (PCR) and sequencing, mutations in unidentified genes, or epigenetic modifications (Carpten et al., 2002; Cetani et al., 2004; Bradley et al., 2005b; Bradley & Thakker, 2006). Approximately 55% of reported germline *CDC73* mutations are associated with HPT‐JT, and these comprise: 60% frameshift, 26% nonsense, and 3% loss of initiator methionine mutations that are predicted to result in parafibromin truncation or loss of protein transcription; 5% missense; and 5% splice site mutations (Newey, Bowl, Cranston, & Thakker, 2010). Of the remaining 45% of germline *CDC73* mutations, 21% are reported from patients with FIHP, and of these 50% are frameshift insertion/deletions, 29% are missense, and 21% are splice site mutations; 15% are reported from patients with sporadic PC, and of these 50% are frameshift insertion/deletions, 40% are nonsense, and 10% are missense mutations; 6% are reported from patients with sporadic adenomas, and of these 50% are missense and 50% are nonsense mutations; and 3% are reported from patients with sporadic ossifying fibromas of the jaw and all of these are frameshift insertion or deletions (Newey et al., 2010). Moreover, the same germline *CDC73* mutations may be associated with HPT‐JT, FIHP, and sporadic PC in different patients; for example, the c.679_680insAG, p.Arg227LysfsX31 mutation has been reported to occur in patients with HPT‐JT (Figure 2 and Table 3), FIHP (Figure 2 and Table 4) and sporadic PC (Figure 2 and Table 5), and the c131+1G > A mutation has been reported to occur in patients with HPT‐JT and FIHP (Tables 3 and 4) (Carpten et al., 2002; Shattuck et al., 2003b; Cetani et al., 2004; Simonds et al., 2004; Bradley et al., 2005a; Newey et al., 2010). Thus, there is a lack of genotype–phenotype correlation and the underlying mechanisms for this variability remain to be elucidated (Howell et al., 2003; Shattuck et al., 2003b; Thakker, 2016).

**Table 4:** Summary of *CDC73*, *MEN1*, and *CASR* mutations associated with familial isolated primary hyperparathyroidism

**Mutation** a	**Exon/intron**	**Codon** b	**Predicted effect** c	**Type** d	**Original designation**	**References**
***CDC73***						
c.61_64del	Exon 1	21	p.Lys21GlufsX4	S^1^	c.61_64del4	Kelly et al. (2006)
c.62_66del	Exon 1	21	p.Lys21ArgfsX43	G	62–66del	Mizusawa et al. (2006)
c.70_73del	Exon 1	24	p.Glu24X	S^2^	70–73del	Mizusawa et al. (2006)
c.95_102del	Exon 1	32	p.Trp32X	S^2^	95–102del	Mizusawa et al. (2006)
c.128G > A	Exon 1	43	p.Trp43X	S	128G→A	Carpten et al. (2002)^q^
c.131+1G > A	Intron 1		splice [d]^n^	G	IVS1+1G > A	Cetani et al. (2004)
c.131+1G > A	Intron 1		splice [d]^n^	G	IVS1+1 g→a	Bradley et al. (2005a)
c.140_144del	Exon 2	47	p.Lys47ArgfsX17	G^o^ ^,1^	c.140_144del5	Kelly et al. (2006)
c.157G > T	Exon 2	53	Glu53X	G	c.157G > T (Glu53X)	Kong et al. (2014)
c.188T > C	Exon 2	63	p.Leu63Pro	G	c.188T > C	Newey et al. (2010)
c.191T > C	Exon 2	64	p.Leu64Pro	G	191T→C	Villablanca et al. (2004)
c.194dup	Exon 2	65	p.Asn65LysfsX2	G	194dupA	Takeuchi et al. (2015)
c.205dup	Exon 2	69	p.Leu69ProfsX13	G^o^	c.205dupC	Pichardo‐Lowden et al. (2011)^e^
c.237+1G > C	Intron 2		splice [d]^n^	G	IVS2+1G→C	Villablanca et al. (2004)^n^ ^(rt)^
c.253_258del	Exon 3	85	p.Val85_Val86del	G	c.252_257del6	Pazienza et al. (2013)
c.272G > C	Exon 3	91	p.Arg91Pro	G^o^	Arg91Pro	Zhang et al. (2012)^f^
c.284T > C	Exon 3	95	p.Leu95Pro	S^o^ ^,3^	c.284T > C	Yu et al. (2015)^g^
c.293T > C	Exon 3	98	p.Leu98Pro	G	c.293T > C exon 3	Bricaire et al. (2013)^n^ ^(ut)^
c.307+1G > A	Intron 3		splice [d]	G^o^	IVS3+1 G > A	Kong et al. (2014)
c.308–9T > A	Intron 3		splice [vus]	G	c.308–9T > A intron 3	Bricaire et al. (2013)^n^ ^(rt,jt?)^
c.343G > T	Exon 4	115	p.Glu115X	G^o^	c.343G > T	Guarnieri et al. (2008)^h^
c.356del	Exon 4	119	p.Gln119ArgfsX14	G^o^ ^,3^	356delA	Bradley et al. (2006)^g^
c.415C > T	Exon 5	139	p.Arg139X	G^o^	c.415C > T	Guarnieri et al. (2008)^h^^,^ i ^,^ n ^(rt,ut)^
c.483_486del	Exon 6	162	p.Glu162GlyfsX39	G	c.481_484delAAAG exon 6	Bricaire et al. (2013)^n^ ^(ut,jt?)^
c.505C > T	Exon 6	169	p. Gln169X	G	c.505C > T	Ghemigian et al. (2013)
c.520_523del	Exon 7	174	p.Ser174LysfsX27	G^o^	c.518_521delTCTC	Guarnieri et al. (2008)^h,i,n(rt,ut)^
c.520_523del	Exon 7	174	p.Ser174LysfsX27	G^2^	518–521del	Mizusawa et al. (2006)
c.664C > T	Exon 7	222	p.Arg222X	G	R222X	Khadilkar et al. (2015)
c.664C > T	Exon 7	222	p.Arg222X	G	c.664 C > T (Arg222X)	Kong et al. (2014)
c.679_680insAG	Exon 7	227	p.Arg227LysfsX31	G^o^	679_680insAG	Simonds et al. (2004)^n^ ^(lip)^
c.685_688del	Exon 7	229	p.Arg229TyrfsX27	G^o^	685delAGAG	Guarnieri et al. (2006)^n^ ^(rt,ut)^
c.745dup	Exon 8	249	p.Ile249AsnfsX18	G	745 dup 1 bp	Bradley et al. (2006)
Gross deletion				G	c.237‐?_308‐?del exon 3	Bricaire et al. (2013)^n^ ^(rt,jt?)^
Gross deletion				G	c.131 ?_308‐?del exons 2–3	Bricaire et al. (2013)
Gross deletion				G^o^	Deletion exon 1–10	Korpi‐Hyovalti et al. (2014)^n^ ^(rt)^
***MEN1***						
c.13_15delinsACGCT	Exon 2	5	p.Ala5ThrfsX115	G	13insACGCTdelGCC	Cardinal et al. (2005)^i^
c.249_252del	Exon 2	85	p.Ile85SerfsX33	G	249del4	Karges et al. (2000)
c.255_256insCAGTGGCCGACCTGTCTAT	Exon 2	86	p.Ile86GlnfsX37	G	2543ins18	Bergman et al. (2000)^i^, ^j^
c.255_256insCAGTGGCCGACCTGTCTAT	Exon 2	86	p.Ile86GlnfsX37	G	c.255_256insCAGTGGCCGACCTGTCTAT	Warner et al. (2004)^i^
c.255_256insCAGTGGCCGACCTGTCTAT	Exon 2	86	p.Ile86GlnfsX37	G	255ins19	Cardinal et al. (2005)^i^
c.334G > C	Exon 2	112	p.Val112Leu	G	L112V	Villablanca et al. (2002)
c.458A > T	Exon 3	153	Asp153Val	G	D153V	Pannett et al. (2003)
c.532_535del	Exon 3	178	p.Ser178ArgfsX6	G	codon 177–178(delGTCT)	Pannett et al. (2003)
c.551T > A	Exon 3	184	p.Val184Glu	G	V184E	Fujimori et al. (1998)
c.590C > T	Exon 3	197	p.Thr197Ile	G	590C > T	Warner et al. (2004)^i^
c.590C > T	Exon 3	197	p.Thr197Ile	G	590C→T	Cardinal et al. (2005)^i^
c.600_601dup	Exon 3	201	p.Lys201ThrfsX24	G	711dupCA	Wautot et al. (2002)^k^
c.654G > T^p^	Exon 3	218		G	codon 219 (CGG→CGT)	Dwarakanathan, Zwart and Oathus (2000)
c.659G > T	Exon 4	220	p.Trp220Leu	G	Trp220Leu	Hannan et al. (2008)
c.673G > A	Exon 4	225	p.Gly225Arg	G	G225R (GGA→AGA)	Mizusawa et al. (2006)
c.722G > T	Exon 4	241	p.Cys241Phe	G	C240F	Wautot et al. (2002)^k^
c.763G > A	Exon 4	255	p.Glu255Lys	G	E255K	Teh et al. (1998)
c.779A > C	Exon 4	260	p.Gln260Pro	G	Q260P	Kassem, Kruse, Wong, Larsson and Teh (2000)
c.784‐9G > A	Intron 4		splice [vus]	G	IVS4 ‐9G→A	Cetani et al. (2006)^n^ ^(rt,lip,tn)^
c.800T > C	Exon 5	267	p.Leu267Pro	G	910T→C	Poncin et al. (1999)
c.824G > T	Exon 5	275	p.Arg275Met	G	c.824G > T	Nagamura et al. (2012)
c.824+1G > A	Intron 5		splice [d]	G	IVS5 +1G→A	Cetani et al. (2006)^n^ ^(lip)^
c.914G > A	Exon 7	305	p.Gly305Asp	G	G305D	Honda et al. (2000)
c.1021T > C	Exon 7	341	p.Trp341Arg	G	c.T1021C: p.W341R	Isakov et al. (2013)
c.1021T > C	Exon 7	341	p.Trp341Arg	G	W341R	Wautot et al. (2002)^k^
c.1049+2_1049+5del	Intron 7		splice [vus]	G	codon 350 (delGAgt)	Pannett et al. (2003)
c.1051T > A	Exon 8	351	p.Tyr351Asn	G	Tyr351Asn	Hannan et al. (2008)
c.1059C > A	Exon 8	353	p.Tyr353X	G	Y353X	Shimizu et al. (1997)
c.1058_1060del	Exon 8	353	p.Tyr353del	G	1057‐1059delACT	Warner et al. (2004)^i^
c.1058_1060del	Exon 8	353	p.Tyr353del	G	1057‐1060delACT	Cardinal et al. (2005)^i^
c.1069G > C	Exon 8	357	p.Asp357His	G	D357H	Wautot et al. (2002)^k^
c.1087_1089del	Exon 8	363	p.Glu363del	G	E363del	Miedlich, Lohmann, Schneyer, Lamesch and Paschke (2001)
c.1096G > T	Exon 8	366	p.Glu366X	G	Q366X	Takami et al. (2000)
c.1190_1193del	Exon 9	397	p.Thr397ArgfsX47	G	1298del4	Wautot et al. (2002)^k^
c.1231G > C	Exon 9	411	p.Ala411Pro	G	A411P	Pannett et al. (2003)
c.1241_1243del	Exon 9	414	p.Leu414del	G	1350del3	Sato et al. (1998)
c.1241_1243del	Exon 9	414	p.Leu414del	G	1350del3	Ohye et al. (1998)
c.1252G > C	Exon 9	418	p.Asp418His	G	1252G > C	Warner et al. (2004)^l^
c.1252G > C	Exon 9	418	p.Asp418His	G	D418H	Cetani et al. (2006)^n^ ^(tn)^
c.1343_1353del	Exon 9	448	p.Glu448AlafsX79	G	1452deL11	Wautot et al. (2002)^k^
c.1350+1G > A	Intron 9		splice [d]	G^o^	IVS9 +1G > A	Carrasco et al. (2004)^q^
c. 1373_1376del	Exon 10	458	p.Val458AlafsX100	G	1483del4	Takami et al. (2000)
c.1382_1404del	Exon 10	461	p.Glu461GlyfsX62	G	1486del23	Wautot et al. (2002)^k^
c.1546dup	Exon 10	516	p.Arg516ProfsX15	G	1546‐1547insC	Warner et al. (2004)
c.1548del	Exon 10	516	p.Lys517SerfsX42	G	1658delG	Villablanca et al. (2002)
c.1676del	Exon 10	559	p.Lys559ArgfsX3	G	1785delA	Cetani et al. (2002)
Gross deletion				G	gross deletion	Cebrian et al. (2003)
***CASR***						
c.299C > T	Exon 3	100	p.Thr100Ile	G	T100I	Warner et al. (2004)^n^ ^(hca)^
c.476T > C	Exon 3	159	p.Leu159Pro	G	L159P	Simonds et al. (2002)^n^ ^(hca,uccr)^
c.658C > T	Exon 4	220	p.Arg220Trp	G	R220W	Simonds et al. (2002)^n^ ^(hca,uccr)^
c.748G > A	Exon 4	250	p.Glu250Lys	G	E250K	Simonds et al. (2002)
c.802_812del	Exon 4	268	p.Val268GlnfsX6	G	V268del‐11 × 273	Simonds et al. (2002)^n^ ^(hca,hcu)^
c.1006_1008del	Exon 4	336	p.Lys336del	G	K336del	Warner et al. (2004)^n^ ^(hca,hcu)^
c.1949T > C	Exon 7	650	p.Leu650Pro	G	L650P	Warner et al. (2004)^n^ ^(hca)^
c.2065G > A	Exon 7	689	p.Val689Met	G	V689M	Warner et al. (2004)^n^ ^(hca)^
c.2641T > C	Exon 7	881	p.Phe881Leu	G	F881L	Carling et al. (2000)^n^ ^(hca)^
c.2657G > C	Exon 7	886	p.Arg886Pro	G	R886P	Simonds et al. (2002)^n^ ^(hca,uccr)^

a Mutations are numbered in relation to the cell division cycle 73 (*CDC73*), multiple endocrine neoplasia type 1 (*MEN1*), and calcium‐sensing receptor (*CASR*) cDNA reference sequences (GenBank accession number NM_024529.4, NM_130799.2, NM_000388.3, respectively) whereby nucleotide +1 corresponds to the A of the ATG‐translation initiation codon. All mutations were analyzed using the Leiden Open Variation Database (LOVD) Mutalyzer sequence variant nomenclature checker (https://www.lovd.nl/mutalyzer/) and annotated using the Human Genome Variation Society (HGVS) guidelines (https://www.hgvs.org/).

b Codon numbering starts from initiation codon of *CDC73*, *MEN1*, and *CASR* mRNA.

c Predicted effect: splice, splice site mutation; [d] donor splice site; [a] acceptor splice site; [vus] variant of unknown significance.

d Mutation type: G, germline; S, somatic; ND, not defined. Equal superscript numbers represent germline and/or somatic mutations occurring in the same patient.

e It is possible this is a case of HPT‐JT associated with PC since: the patient was diagnosed with three renal cysts, while “a maternal cousin had jaw pain and presumably bone destruction of the jaw, termed a ‘hole in the jaw’.” Furthermore, histological description of the proband's parathyroid gland was consistent with an APA (“…vascular and capsular invasion, but no definitive features of PC were identified”) and disease recurrence on the contra‐lateral side (again with diagnosis of APA) suggests a more malignant behavior.

f All mutation carriers (*n* = 3) of this kindred developed PC.

g Kindred originally reported by Williamson et al., and classified as HPT‐JT by Carpten et al. and FIHP by Bradley et al., and associated with PC by Carpten et al., Bradley et al., and Yu et al. (Williamson et al., 1999; Carpten et al., 2002; Bradley et al., 2005a; Bradley et al., 2006; Yu et al., 2015).

h Reported as a FIHP family, but no information was provided on the pHPT status of the mutation carriers.

Additional clinical details about these kindreds are provided by Corbetta et al. (2010) and Vaira et al. (2012).

i Studies reported by the same group, therefore it is not possible to exclude that equal mutations described in different publications are from the same proband/kindred.

j Mutation was incorrectly reported in the original publication and was posteriorly updated by Warner et al. (2004) and Cardinal et al. (2005).

k The authors collected 165 *MEN1* mutations in patients with MEN1, but seven probands/kindreds exhibited FIHP phenotype (i.e., only pHPT) and were included here.

l In a posterior publication, this mutation was identified by the same group in a kindred with MEN1 syndrome, and it is unclear if there were two different kindreds with the same mutation or if it was an update of the previous kindred (Cardinal et al., 2005).

m Presence in the probands/kindreds of: ^rt^ renal cysts/lesions, and/or ^ut^ uterine tumors (if the presence of renal cysts or uterine tumors was unknown, one “?” was added next to the previous superscripts; ^hjt?^ was added if the absence of jaw tumors was unknown), and/or ^lip^ lipoma, and/or ^tn^ thyroid nodules, and/or ^hca^ hypercalcemia, and/or ^hcu^ hypercalciuria, and/or ^uccr^ urine calcium/creatinine clearance ratio < 0.010 in most of the affected individuals.

n For detailed information of the effect of *CDC73* mutation on splicing please consult Hahn et al. (2009).

o Mutations identified in kindreds with case reports of PC.

p Discordant codon/nucleotide number in the original report. There is no predicted change on the amino acid (p.Arg218 = ), but the authors reported altered RNA splicing caused by this nucleotide change.

q Criteria for diagnosis of PC not reported.

HPT‐JT, hyperparathyroidism‐jaw tumor; PC, parathyroid carcinoma; APA, atypical parathyroid adenoma; FIHP, familial isolated primary hyperparathyroidism; MEN1, multiple endocrine neoplasia type 1.

**Table 5:** Summary of *CDC73* mutations associated with sporadic parathyroid carcinoma

**Mutation** a	**Exon/intron**	**Codon** b	**Predicted effect** c	**Type** d	**Original designation**	**References**
c.13C > T	Exon 1	5	p.Leu5Phe	S	13C > T	Guarnieri et al. (2012)
c.14T > C	Exon 1	5	p.Leu5Pro	S^1^	c.14T > C	Cavaco et al. (2011)
c.16del	Exon 1	6	p.Ser6AlafsX15	ND^2^	16delA	Shattuck et al. (2003b)
c.23_25delinsGT	Exon 1	8	p.Leu8ArgfsX13	S	23TGCG > GTG	Shattuck et al. (2003b)
c.25C > T	Exon 1	9	p.Arg9X	S	R9X	Cetani et al. (2004)
c.32del	Exon 1	11	p.Tyr11SerfsX10	S^3^	c.32delA	Domingues et al. (2012)^e^
c.34_35insCT	Exon 1	12	p.Asn12ThrfsX10	ND	c.34_35insCT	Wang et al. (2012)^j^
c.34_37del	Exon 1	12	p.Asn12SerfsX8	S	34‐37 delAACA	Enomoto et al. (2010)^j^
c.40del	Exon 1	14	p.Gln14ArgfsX7	S	39delC	Shattuck et al. (2003b)
c.42del	Exon 1	15	p.Lys15ArgfsX6	S^4^	c.42delG	Guarnieri et al. (2012)
c.60del	Exon 1	21	p.Lys21ArgfsX5	S^5^	c.60delG	Cetani et al. (2013)
c.64_73del	Exon 1	22	p.Gly22X	ND^6^	60del10	Shattuck et al. (2003b)
c.64G > T	Exon 1	22	p.Gly22X	S^7^	c.64G > T	Cetani et al. (2013)
c.70G > T	Exon 1	24	p.Glu24X	S^8^	70G > T	Shattuck et al. (2003b)
c.70G > T	Exon 1	24	p.Glu24X	S	E24X	Cetani et al. (2007)
c.70G > T	Exon 1	24	p.Glu24X	G	c.70G > T	Serrano‐Gonzalez, Shay, Austin, Maceri and Pitukcheewanont (2016)
c.76del	Exon 1	26	p.Ile26SerfsX11	S	c.76delA	Howell et al. (2003)
c.82_85del	Exon 1	28	p.Gly28SerfsX8	S^9^	82del4	Shattuck et al. (2003b)
c.85del	Exon 1	29	p.Glu29SerfsX8	S^10^	c.85delG	Siu et al. (2011)
c.88_94del	Exon 1	30	p.Phe30GlyfsX5	S	c.88_94delTTCTCCT	Frank‐Raue et al. (2011)^h^ ^(rt)^
c.94_95insTA	Exon 1	32	p.Trp32LeufsX6	S	c.94insTA	Guarnieri et al. (2012)
c.128G > A	Exon 1	43	p.Trp43X	S	c.128G > A	Haven et al. (2007)^f^
c.162C > G	Exon 2	54	p.Tyr54X	S	c.162C > G (Y54X)	Howell et al. (2003)
c.162C > G	Exon 2	54	p.Tyr54X	S^11^	162C > G	Shattuck et al. (2003b)
c.162C > G	Exon 2	54	p.Tyr54X	ND	c.162C > G	Wang et al. (2012)
c.162C > A	Exon 2	54	p.Tyr54X	S^12^	c.162C > A	Cavaco et al. (2011)^g^
c.165C > A	Exon 2	55	p.Tyr55X	S^13^	c.165C > A	Howell et al. (2003)
c.165del	Exon 2	55	p.Tyr55X	S	c.165delC	Howell et al. (2003)
c.165del	Exon 2	55	p.Tyr55X	S	c.165delC	Haven et al. (2007)^f^
c.176C > T	Exon 2	59	p.Ser59Phe	G	c.176C > T	Haven et al. (2007)^f^
c.182T > A	Exon 2	61	p.Leu61X	S	182T > A	Cetani et al. (2007)
c.195dup	Exon 2	66	p.Asn66X	S	195insT	Cetani et al. (2004)
c.197dup	Exon 2	66	p.Asn66LysfsX16	S	195insA	Cetani et al. (2004)
c.226C > T	Exon 2	76	p.Arg76X	S	c.226C > T	Shattuck et al. (2003b)
c.226C > T	Exon 2	76	p.Arg76X	G^1^	c.226C > T	Cavaco et al. (2011)
c.226C > T	Exon 2	76	p.Arg76X	G^10^	c.226C > T	Siu et al. (2011)
c.249del	Exon 3	84	p.Pro84LeufsX25	S^5^	c.248delT	Cetani et al. (2013)
c.260_261del	Exon 3	87	p.Arg87LysfsX3	G	c.260_261delGA	Wang et al. (2012)
c.343G > T	Exon 4	115	p.Glu115X	G	E115X	Cetani et al. (2013)
c.343G > T	Exon 4	115	p.Glu115X	G^7^	E115X	Cetani et al. (2013)
c.375dup	Exon 5	126	p.Arg126ThrfsX5	G	373insA	Shattuck et al. (2003b)
c.415C > T	Exon 5	139	p.Arg139X	G	415C > T	Cetani et al. (2007)
c.415C > T	Exon 5	139	p.Arg139X	G	c.415C > T exon 5	Bricaire et al. (2013)^j^^,^ h ^(ut?,rt?,jt?)^
c.513‐1del	Intron 6		splice [a]^i^	S^13^	IVS6‐1delG	Howell et al. (2003)
c.520_523del	Exon 7	174	p.Ser174LysfsX27	G^12^	c.518_521delTGTC	Cavaco et al. (2011)^g^
c.544dup	Exon 7	182	p.Ile182AsnfsX11	G	c.539_544insA, p.Ile182AsnfsX10	Yu et al. (2015)
c.626_629del	Exon 7	209	p.Lys209ArgfsX9	G	c.626_629delAACA	Wang et al. (2012)^h(rt)^
c.664C > T	Exon 7	222	p.Arg222X	G	664C > T	Shattuck et al. (2003b)
c.664C > T	Exon 7	222	p.Arg222X	G	c.664C > T exon 7	Bricaire et al. (2013)^h^ ^(rt)^
c.679_680insAG	Exon 7	227	p.Arg227LysfsX31	G^11^	679insAG	Shattuck et al. (2003b)
c.685_688del	Exon 7	229	p.Arg229TyrfsX27	G	c.679_682delAGAG	Corbetta et al. (2010)
c.687_688del	Exon 7	229	p.Arg229SerfsX37	S	c.679_680delAG	Corbetta et al. (2010)
c.687_688del	Exon 7	229	p.Arg229SerfsX37	G^4^	c.679_680delAG	Guarnieri et al. (2012)
c.687_688del	Exon 7	229	p.Arg229SerfsX37	G	c.687_688delAG	Wang et al. (2012)
c.687_688del	Exon 7	229	p.Arg229SerfsX37	G	c.687_688delAG	Witteveen et al. (2011)^f^
c.691dup	Exon 7	231	p.Trp231LeufsX36	G	c.692_693insT	Haven et al. (2007)^f^
c.693dup	Exon 7	232	p.Arg232GlufsX35	ND	c.693_694insG	Haven et al. (2007)
c.700C > T	Exon 7	234	p.Arg234X	ND^6^	700C > T	Shattuck et al. (2003b)
c.700C > T	Exon 7	234	p.Arg234X	G	R234X	Cetani et al. (2004)
c.700C > T	Exon 7	234	p.Arg234X	G	234 CGA to TGA	Enomoto et al. (2010)
c.736del	Exon 8	246	p.Ser246ProfsX11	S^9^	732delT	Shattuck et al. (2003b)
c.750del	Exon 8	250	p.Phe250LeufsX7	S^8^	746delT	Shattuck et al. (2003b)
c.1231del	Exon 14	411	p.Gln411ArgfsX17	ND^2^	1230delC	Shattuck et al. (2003b)
Gross deletion				G	Whole gene deletion	Bricaire et al. (2013)^h(ut)^
Gross deletion				G	Whole gene deletion	Caron et al. (2011)
Gross deletion				G^3^	Whole gene deletion	Domingues et al. (2012)^e^

a Mutations are numbered in relation to the cell division cycle 73 (*CDC73*) cDNA reference sequence (GenBank accession number NM_024529.4) whereby nucleotide +1 corresponds to the A of the ATG‐translation initiation codon. All mutations were analyzed using the Leiden Open Variation Database (LOVD) Mutalyzer sequence variant nomenclature checker (https://www.lovd.nl/mutalyzer/) and annotated using the Human Genome Variation Society (HGVS) guidelines (https://www.hgvs.org/).

b Codon numbering starts from initiation codon of *CDC73* mRNA.

c Predicted effect: splice, splice site mutation; [d] donor splice site; [a] acceptor splice site.

d Mutation type: G, germline; S, somatic; ND, not defined. Equal superscript numbers represent germline and/or somatic mutations occurring in the same patient.

e Initially reported as a benign parathyroid adenoma, but later reclassified as PC by Yu et al. (2015).

f In a posterior publication, most of this cohort was updated by Witteveen et al. (2011).

g PC diagnosis disputable since: the tumor recurrence occurred with several cervical nodules of parathyroid tissue (fibrous septae, with low pleomorphism and high proliferative activity); however, during the first surgery, where a typical parathyroid adenoma was removed, the capsule was ruptured, thus raising the possibility of local seeding.

h Presence in the affected patient of: ^rt^ renal cysts/lesions, and/or ^ut^ uterine tumors (if the presence of renal cysts or uterine tumors was unknown, one “?” was added next to the previous superscripts; ^hjt?^ was added if the absence of jaw tumors was unknown).

i For detailed information of the effect of *CDC73* mutation on splicing please consult Hahn et al. (2009).

j Criteria for diagnosis of PC not reported.

PC, parathyroid carcinoma.

### 3.2 Multiple endocrine neoplasia type 1 (MEN1)

MEN1 (MIM# 131100), also known as Wermer's syndrome, is characterized by the occurrence of parathyroid, pancreatic islet, and anterior pituitary tumors (Thakker, 1998). Parathyroid tumors are often the first and the most frequent tumors, and occur in approximately 95% of MEN1 patients (Thakker et al., 2012; Thakker, 2014). Unlike HPT‐JT, many MEN1 patients with parathyroid tumors have multiglandular disease.

#### 3.2.1 MEN1

MEN1 is an autosomal dominant disease, due to germline mutations of the *MEN1* gene located on chromosome 11q13. *MEN1* encodes the protein menin, which has roles in transcriptional regulation, genome stability, cell division, and proliferation. These roles have been identified by studying menin interactions with proteins. Thus, menin's roles in: transcriptional regulation involves interactions with Jun‐mediated transcriptional activation, nuclear factor‐kappaB (NF‐κB)‐mediated transcriptional activation, small body size homolog (sma, C. elegans), and mothers against decapentaplegic homolog (mad, Drosophila) (SMAD) family members to inhibit transforming growth factor‐b (TGF‐b) and bone morphogenetic protein‐2 (BMP‐2) signaling, and forkhead transcription factor checkpoint suppressor 1 (CHES1) in an S‐phase checkpoint pathway response to DNA damage; genome stability entails interactions with subunit of replication protein (RPA2) and Fanconi anemia complementation group D2 protein (FANCD2) that is involved in DNA repair; cell division includes interactions with nonmuscle myosin II‐A heavy chain (NMHC II‐A), glial fibrillary acidic protein (GFAP) and vimentin; and in proliferation, the reported interactions are with non‐metastatic cells 1 protein (NME1) and activator of S‐phase kinase (ASK) (Thakker, 2014). More than 90% of tumors from MEN1 patients will have LOH of *MEN1*, with loss of menin expression, consistent with a tumor suppressor role for *MEN1* (Thakker et al., 1989; Lemos & Thakker, 2008). Approximately 10% of MEN1 patients harbor de novo mutations and 10%–15% may develop a non‐familial form (i.e., sporadic) (Trump et al., 1996; Bassett et al., 1998). To date >1,800 *MEN1* mutations have been reported and ∼40% of these mutations are frameshift, followed by ∼25% nonsense, ∼20% missense mutations, and ∼10% splice site mutations (Lemos & Thakker, 2008; The Universal Mutation Database, 2017). Therefore, >70% of mutations are predicted to lead to truncated, and thus inactivated, forms of menin, with the majority of missense mutations resulting in the mutant menin being targeted to the proteasome, thereby reducing its ability to act as a tumor suppressor (Lemos & Thakker, 2008; Lemos et al., 2009). However, 5%–10% of MEN1 cases do not harbor mutations in the *MEN1* gene (Bassett et al., 1998; Lemos & Thakker, 2008). *MEN1* germline mutations have been reported in patients with hereditary and sporadic MEN1, and in FIHP and somatic *MEN1* mutations are detected in approximately 20% of sporadic parathyroid tumors (Thakker, 2010).

PC rarely occurs in patients with MEN1. To date only 13 PC cases, of whom eight (> 60%) had local invasion or metastasis, have been reported in association with MEN1 (Table 6); one of these patients developed multiglandular PC, and in the remainder of patients, the PC was associated with multiple adenomatosis or hyperplasia. Four (30%) of these MEN1 patients presented with hypercalcemic crisis (median total calcium 15.7 mg/dl, that is, 3.9 mmol/l, and PTH 309.5 pg/ml) at a mean age of 50 years old. *MEN1* germline mutations were reported in six (>45%) of these patients, and comprised one nonsense, three frameshifting with premature truncations, and two missense mutations (Sato et al., 2000; Clerici et al., 2001; Tham et al., 2007; Juodele et al., 2011; Christakis et al., 2016). Somatic genetic abnormalities in these PCs were not reported.

**Table 6:** Parathyroid carcinoma in multiple endocrine neoplasia

Gender Age	1^st^ manifestation	Calcium^a^ (mg/dL)	PTH^b^ (pg/mL)	Associated conditions	Mutation^c^	Predicted effect	Notes	References
**MEN1**								
Male 52yr	Hypercalcaemic crisis	16.4	154.3	Pituitary adenoma	ND		Uniglandular PC Local recurrence and chest wall metastasis No MEN1 family history	[Wu, et al., 1992]
Female 51yr	Thyroid mass Mild hypercalcaemia	10.7	ND		c.734delC^d^^,^ ¶	p.Pro245LeufsX36	Uniglandular PC¥ and 3 PAs No metastasis reported No MEN1 family history	[Sato, et al., 2000]
Male 35yr	Hypercalcaemic crisis	15.7	1,888	Pancreatic gastrinoma Gastric carcinoid Lipomas	NR		Ectopic PC and 3 PAs Mediastinal metastasis No MEN1 family history	[Dionisi, et al., 2002]
Male 32yr	Hypercalcaemic crisis	14.8	264	Pancreatic gastrinoma Islet cell tumour Adrenal hyperplasia Cushing's syndrome	No		Uniglandular PC No metastasis reported Mother with pHPT and daughter with hypoglycaemia	[Agha, et al., 2007]
Female 65yr	Hypercalcaemic crisis	15.6	355	Non‐functioning islet cell tumour Macroprolactinoma	No		Uniglandular PC Mediastinal metastasis No MEN1 family history	[Agha, et al., 2007]
Female 53yr	Moderate hypercalcaemia	13.4	1,354	Gastrinoma Non‐functioning pituitary adenoma Adrenal nodularity	c.1406_1413dup8^e^^,^ ¶	p.Gly472SerfsX90	Uniglandular PC and 1 PA Recurrent laryngeal nerves and trachea invasion No MEN1 family history	[Shih, et al., 2009]
Female 44yr	Mild hypercalcaemia Cervical pain	10.6	68	Acromegaly Pancreatic tumour	NR		Uniglandular PC Lung metastasis No MEN1 family history	[Kalavalapalli, et al., 2010]
Male 50yr	Moderate hypercalcaemia	≈12	204	Malignant gastrinoma Non‐functioning pituitary adenoma	c.549G>T^f^^,^ ¶	p.Trp183Cys	Uniglandular PC and 1 hyperplasic gland No metastasis reported Daughter carries c.549G>T	[del Pozo, et al., 2011]
Female 39yr	Cervical mass	13.4	323	Microprolactinoma Malignant insulinoma Adrenal tumour Lipomas	c.129_130insA^g^^,^ ¶	p.Val44SerfsX73	Multiglandular PC Thyroid invasion and metastasis Father died from complications of refractory gastric ulcer (gastrinoma?) Son carries c.129_130insA	[Juodele, et al., 2011]
Female 59yr	Moderate hypercalcaemia	12.7	248.2	2 pituitary microadenomas Adrenal nodule	NR		Uniglandular PC Thyroid invasion	[Lee, et al., 2014]
Male 62yr	Moderate hypercalcaemia	12.4	127.3	Gastrinoma Bronchial carcinoid Adrenal nodule	ND		Uniglandular PC and 1 hyperplasic gland Left recurrent laryngeal nerve invasion (and possibly oesophagus) Family history of MEN1	[Singh Ospina, et al., 2016]
Male 54yr	Moderate hypercalcaemia	10.5	42	Pancreatic endocrine tumour Bronchial carcinoid	c.703G>A^h^^,^ ¶	p.Glu235Lys	Uniglandular PC and 2 hyperplasic glands No metastasis reported	[Christakis, et al., 2016]
Male 55yr	Hypercalcemia Cervical mass	13.8	673.1	Pancreatic endocrine tumour Pituitary tumour Adrenal tumour	c.1378C>T^i^^,^ ¶	p.Arg460X	Uniglandular PC$ and 3 hyperplasic glands No metastasis reported	[Christakis, et al., 2016]
**MEN2**								
Male 47yr	Moderate hypercalcaemia	13.6	443	Medullary thyroid carcinoma	c.1901G>A^j^^,^ ¶	p.Cys634Tyr	Unknown primary location of PC Bone metastasis No MEN2 family history	[Jenkins, et al., 1997]
Male 49yr	Severe hypercalcaemia *Osteitis fibrosa cystica*	15.1	1,399	Medullary thyroid carcinoma	No		Unknown primary location of PC Lung metastasis Son was diagnosed with pHPT	[Alfaro, et al., 2002]
Male 54yr	Asymptomatic	9.2	57.5	Pheochromocytoma	c.1852T>C^k^^,^ ‡	p.Cys618Arg	Uniglandular PC Cervical lymph node metastasis Family history of MEN2	[Posada‐Gonzalez, et al., 2014]

^a^Total serum calcium reference limits: 8.8–10.5 mg/dL (converted to commonly used units).

^b^Parathyroid hormone (PTH) serum reference limits 10–65 pg/mL (converted to commonly used units).

^c^Mutations are numbered in relation to the multiple endocrine neoplasia type 1 (*MEN1*) and rearranged during transfection (*RET*) cDNA reference sequences (GenBank accession number NM_130799.2 and NM_020975.4, respectively) whereby nucleotide +1 corresponds to the A of the ATG‐translation initiation codon. All mutations were analysed using the Leiden Open Variation Database (LOVD) Mutalyzer sequence variant nomenclature checker (http://www.lovd.nl/mutalyzer/) and annotated using the Human Genome Variation Society (HGVS) guidelines (http://www.hgvs.org/).

Reported originally as: ^d^ c.842delC, ^e^ c.1406_13dup8;^f^W183; ^g^ c.129insA; ^h^ c.703G>A; ^i^ c.1378C>T; ^j^ p.C634Y;^k^ Cys618Arg.

^¶^Germline mutation;^‡^germline or somatic origin not defined (possibly germline, since its identification led to the prophylactic thyroidectomy, where the PC was incidentally found).

^¥^Diagnosis of PC based on capsular invasion, mitoses in parenchymal cells, and nuclear polymorphism. *MEN1* mutation (c.734delC) was not identified in the 4 family members screened.

^$^Diagnosis of PC based on capsular invasion, fibrosis, cellular pleomorphism, dense fibrotic bands, and angulated parathyroid cell nests.

PC, parathyroid carcinoma; PA, parathyroid adenoma; pHPT, primary hyperparathyroidism; MEN1, multiple endocrine neoplasia type 1; MEN2, multiple endocrine neoplasia type 2; ND, not done; NR, not reported.

#### 3.3 Multiple endocrine neoplasia type 2 (MEN2)

MEN2, also known as Sipple's syndrome, comprises three variants referred to as MEN2A (MIM# 171400), MEN2B (also called MEN3) (MIM# 162300), and medullary thyroid carcinoma (MTC) (MIM# 155240). MEN2A is characterized by occurrence of MTC, pheochromocytoma, and parathyroid tumors, which occur in >99%, ∼40%, and ∼30% of patients, respectively (Howe, Norton, & Wells, 1993). MEN2B is characterized by occurrence of MTC and pheochromocytoma in association with mucosal neuromas, medullated corneal fibers, intestinal autonomic ganglion dysfunction, and a Marfanoid habitus (Thakker, 1998). In patients with MTC‐only, MTC is the sole manifestation.

#### 3.3.1 RET

MEN2A, MEN2B, and MTC‐only are due to activating mutations of the *RET* gene, located on chromosome 10q11.21 (Mathew et al., 1987; Simpson et al., 1987; Donis‐Keller et al., 1993; Mulligan, et al., 1993). The *RET* gene encodes a receptor tyrosine‐protein kinase involved in cell proliferation, neuronal navigation, cell migration, and cell differentiation following binding of glial cell‐derived neurotrophic factor ligands. RET signaling has critical roles in kidney organogenesis and formation of neural crest‐derived lineages, and RET can also modulate cell adhesion via caspase cleavage and cell migration in an integrin‐dependent manner. Moreover, in the absence of ligand, RET can also trigger apoptosis via intracellular caspase cleavage of the receptor (Mehlen & Thibert, 2004; Plaza‐Menacho, Mologni, & McDonald, 2014). There is a genotype–phenotype correlation between *RET* mutations and MEN2A, MEN2B, and MTC‐only, with: the majority of MEN2A patients having *RET* germline mutations involving codons 609, 611, 618, or 620 of exon 10, or codon 634 of exon 11; MEN2B patients having mutations of codon 918; and MTC‐only patients having mutations involving codons 618, 790, 791, or 804 (Raue & Frank‐Raue, 2012). To date three PC cases have been reported in association with MEN2A (Table 6). All patients were men and all had PC metastasis at diagnosis. *RET* mutations were identified in two of these patients, and these comprised a c.1852T > C, p.Cys618Arg mutation, whose germline or somatic origin was not defined, and a germline c.1901G > A, p.Cys634Tyr mutation. The metastatic PC from the patient with the *RET* Cys634Tyr mutation had additional somatic genetic abnormalities involving LOH at loci from chromosomes 1, 2, 3p, 13q, and 16p (Jenkins et al., 1997).

#### 3.4 Familial isolated primary hyperparathyroidism (FIHP)

FIHP (MIM♯ 145000), is an autosomal dominant disorder, and to date >100 families with FIHP have been reported (Simonds et al., 2002; Pannett et al., 2003; Pontikides et al., 2014). The prevalence of FIHP has been estimated to be ∼1% of all pHPT cases, with an age at diagnosis of 40 years old (Simonds et al., 2002). Patients with FIHP more frequently present with severe hypercalcemia when compared with MEN1 patients or sporadic pHPT patients, and the provisional diagnosis of FIHP may, in ∼20% of patients, be reclassified as HPT‐JT, MEN1, or familial hypocalciuric hypercalcemia (FHH) following development of syndromic manifestations (Simonds et al., 2002; Pontikides et al., 2014). Furthermore, FIHP predisposition for PC is particularly high for *CDC73* mutation carriers (Simonds et al., 2002; Pontikides et al., 2014).

#### 3.4.1 CDC73

*CDC73* mutations occur in 8% of FIHP patients (Pontikides et al., 2014). The majority of *CDC73* germline mutations associated with FIHP are frameshift or nonsense, predicting premature truncation of parafibromin, thereby supporting a tumor suppressor function (Table 4 and Figure 2A and B).

#### 3.4.2 MEN1

*MEN1* mutations occur in 20% of FIHP patients (Pontikides et al., 2014). LOH, particularly at the 11q13 region, is a common finding in FIHP tumor samples. *MEN1* germline mutations have been reported in 42 FIHP families, and ∼40% of these were missense, ∼30% were frameshift, and 5% were nonsense mutations (Table 4) (Lemos & Thakker, 2008). Interestingly, FIHP patients, in contrast to MEN1 patients, have a significantly lower prevalence of frameshift/nonsense *MEN1* mutations (∼35% vs. ∼65%).

#### 3.4.3 CASR

The calcium sensing receptor (*CASR*) gene, locate on chromosome 3q13.33, encodes a G‐protein coupled receptor that is predominantly expressed in the parathyroids and kidneys, where it respectively regulates PTH secretion and renal tubular calcium reabsorption appropriate to the prevailing calcium concentration (Thakker, 2004). The CaSR is also expressed in other tissues where its function remains to be elucidated (Thakker, 2004). *CASR* mutations occur in 2% of FIHP patients (Pontikides et al., 2014). To date 10 kindreds with *CASR* mutations associated with FIHP have been reported, and all of them had heterozygous *CASR* mutations that were predicted to be inactivating (Table 4). However, no PC case has been reported in any individual from these FIHP kindreds. FIHP patients with MEN1 and *CASR* mutations are generally younger and have multiglandular disease, whereas patients with *CDC73* mutations have a disproportionally high prevalence of PC (Warner et al., 2004; Iacobone et al., 2007).

#### 3.4.4 GCM2

Recently, activating mutations of the glial cells missing 2 (*GCM2*) gene, located on chromosome 6p24.2, have been reported in FIHP patients (Guan et al., 2016). *GCM2* encodes a protein that acts as a transcription factor regulating parathyroid development and may also act to regulate the effect of calcium on PTH expression and secretion by parathyroid cells (Kamitani‐Kawamoto et al., 2011; Han, Tsunekage, & Kataoka, 2015).

### 3.4.5 Other Genes

*MEN1*, *CDC73*, *CASR*, and *GCM2* mutations may not be found in over 60% of FIHP patients (Pontikides et al., 2014). Interestingly, one study has reported a 1.7 Mb interval of significant genetic linkage for FIHP on chromosome 2p13.3‐14, although conservative mutations involving the protein phosphatase 3 regulatory subunit B alpha (*PPP3R1*) and prokineticin receptor 1 (*PROKR1*) genes, which are in this interval, were not identified (Warner et al., 2006).

## 4 SPORADIC AND NON‐HEREDITARY PARATHYROID CARCINOMA

**Figure 3:**
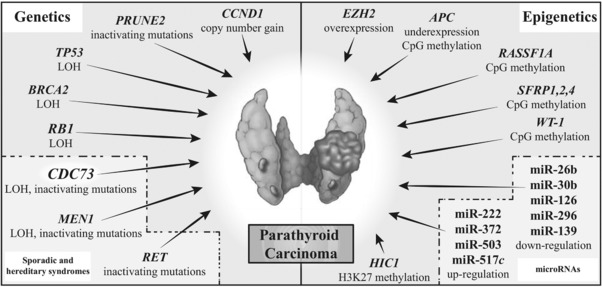
Molecular mechanisms of parathyroid carcinoma. LOH, loss of heterozygosity

Sporadic and non‐hereditary PC may be associated with abnormalities of tumor suppressor genes and oncogenes, similarly to those causing hereditary syndromic forms of PC, and these include *CDC73* and *MEN1* mutations (Figure 3). However, sporadic and non‐hereditary PCs may be associated with abnormalities of other genes, which include retinoblastoma 1 (*RB*), tumor protein P53 (*TP53*), cyclin D1 (*CCND1*), enhancer of zeste 2 polycomb repressive complex 2 subunit (*EZH2*), adenomatous polyposis coli (*APC*), glycogen synthase kinase 3 beta (*GSK3B)*, and prune homolog 2 (*PRUNE2*). In addition, epigenetic abnormalities and microRNAs (miRNAs) may also be involved (Figure 3). These will be reviewed.

### 4.1 CDC73

Approximately 40% of *CDC73* mutations identified in patients with sporadic PC are germline mutations (Table 5 and Figures 2 and 3), and of these *CDC73* mutations ∼65% occur in exons 1, 2, and 7, and the majority are frameshift or nonsense mutations, resulting in premature protein truncation and loss of protein function (Table 5) (Marsh et al., 2007; Newey et al., 2010). Moreover, a non‐random gain of mutated *CDC73* alleles has been reported in PC, and this suggests that aberrant *CDC73* expression may also be important in the pathogenesis of PC (Yu et al., 2015). For example, a recent study reported that 4 of 22 (∼20%) PCs had a 3–5 copy number gain of mutant alleles, with three of these four PCs also having loss of the wild‐type *CDC73* allele through focal deletion or loss of the whole chromosome arm (Yu et al., 2015).

The identification of germline *CDC73* mutations in patients with apparently sporadic PC is important, as it indicates that the patient and relatives are at risk of developing HPT‐JT‐associated tumors. Such germline *CDC73* mutations are reported to occur in 20%–40% of patients with apparently sporadic PC, and somatic *CDC73* mutations have been reported to occur in ∼40%–100% of apparently sporadic PCs (Table 5 and Figure 2) (Howell et al., 2003; Shattuck et al., 2003b; Cetani et al., 2004; Guarnieri et al., 2012). Moreover, LOH involving the *CDC73* locus, on chromosome 1q31.2, is reported to occur in 50%–55% of sporadic PCs, and loss or reduced nuclear expression of parafibromin, detected by immunohistochemical (IHC) analysis, has been reported in >70% of PCs (Haven, van Puijenbroek, Karperien, Fleuren, & Morreau, 2004; Tan, et al., 2004; Cetani et al., 2007; Juhlin, et al., 2007; Yip et al., 2008). In contrast, germline *CDC73* mutations were not found in patients with sporadic PAs, or in patients with hyperplastic parathyroids, and somatic *CDC73* mutations and LOH of chromosome 1q have been reported to occur in <5% and <5%–10% of sporadic PAs, respectively (Carpten et al., 2002; Howell et al., 2003; Cetani et al., 2004; Krebs, Shattuck, & Arnold, 2005; Yip et al., 2008).

The absence of parafibromin nuclear staining, detected by IHC analysis, has been reported to occur in 15% of APAs, and <5%–20% of PAs, and it seems that the ability to distinguish between PC, APA, and PA using parafibromin IHC appears to be lower than *CDC73* mutational analysis (Tan et al., 2004; Gill et al., 2006; Juhlin, et al., 2006; Cetani et al., 2007; Guarnieri et al., 2012; Cetani et al., 2013; Hu, Liao, Cao, Gao, & Zhao, 2016). However, one study has reported that the absence of parafibromin nuclear staining, detected by IHC analysis has a sensitivity of ∼70% and specificity of 95% for diagnosis of PCs (Hu et al., 2016). These findings indicate that *CDC73* mutations are major driver mutations in the etiology of PCs.

### 4.2 MEN1

About 40%–50% of PCs have LOH of chromosome 11q, which is the location of the *MEN1* gene, and >35% of PCs have combined LOH of 11q and 1q, which is the location of *CDC73* (Figure 3). Combined LOH of 11q and 1q is rarely observed in PAs, and these findings suggest that *MEN1* may be involved in PC pathogenesis (Dwight et al., 2000; Haven et al., 2004). In addition, somatic *MEN1* mutations have been reported to occur in <15% PCs, in contrast to the higher frequencies of 35% and >45% of somatic *MEN1* mutations and LOH involving chromosome 11 in sporadic PAs, respectively (Haven et al., 2007; Newey et al., 2012). Thus, the involvement of the *MEN1* gene is likely to be a rare occurrence in PCs.

### 4.3 RB1

The retinoblastoma 1 (*RB1*) tumor suppressor gene, located on chromosome 13q14.2 encodes a protein (RB1) that is a negative regulator of the cell cycle. The active hypophosphorylated form of RB1 binds to the transcription factor E2 promoter binding factor 1 (E2F1) and leads to cell cycle arrest, whereas the phosphorylated form of RB1 allows dissociation from E2F1 and leads to transcription of E2F1 target genes that are involved in cell progression through G1 phase of the cell cycle (Asghar, Witkiewicz, Turner, & Knudsen, 2015). RB1 also maintains chromatin structure by stabilizing constitutive heterochromatin through stabilization of histone methylation (Gonzalo et al., 2005; Dyson, 2016). The *RB1* gene has been implicated in the pathogenesis of PC, as allelic loss of *RB1* has been observed in ∼30%–100% of PCs and decreased RB1 expression has been reported in >85% of PCs (Figure 3) (Cryns et al., 1994b; Dotzenrath et al., 1996; Szijan et al., 2000). This contrasts with the low rate (i.e., <5%) of *RB1* allelic loss in PAs, and no loss of RB1 expression (Cryns et al., 1994b). However, no *RB1* somatic mutations have been identified in PCs, although *RB1* allelic loss has been reported to be associated with PC recurrence and aggressive PA (Pearce et al., 1996; Shattuck et al., 2003a).

### 4.4 TP53

Tumor protein P53 (*TP53*), is a tumor suppressor gene, which is located on chromosome 17p13.1 and encodes a protein (p53) that is a transcription factor whose level and post‐translational modification state are altered in response to cellular stress to induce growth arrest or apoptosis. Activated p53 suppresses cellular transformation by inducing growth arrest, apoptosis, DNA repair, and differentiation in damaged cells (Brosh & Rotter, 2009). *TP53* allelic loss has been reported in 1 of 3 PCs studied (Figure 3), whereas *TP53* overexpression has been observed in ∼10% of PAs (Cryns, Rubio, Thor, Louis, & Arnold, 1994a; Kishikawa et al., 1999). However, a somatic *TP53* missense mutation (c.743G > A, p.Arg248Gln) has been reported in anaplastic PC cells, but not in differentiated PC cells, suggesting an association between this *TP53* mutation and anaplastic transformation (Hakim & Levine, 1994; Tamura et al., 2009). The *TP53* Arg248 residue is part of DNA binding domain (DBD) that interacts directly with the minor groove of DNA, and the p.Arg248Gln mutation is reported to result in the loss of DNA binding via the DBD (Ng et al., 2015). Interestingly, such *TP53* mutations affecting Arg248 are reported to be present in ∼4% of all cancers (Petitjean et al., 2007).

### 4.5 CCND1

Cyclin D1 (*CCND1*), also known as parathyroid adenoma 1 (*PRAD1*), is an oncogene located on chromosome 11q13.3, that encodes cyclin D1, a 295‐amino acid protein that is a component of the cyclin D1‐cyclin‐dependent kinase 4 (CDK4) complex that phosphorylates RB1 and thus inhibits the actions of RB1 in regulating G1/S transition (Arnold et al., 1992). Overexpression of cyclin D1 occurs in ∼65%–90% of PCs, but in <40% of PAs and ∼60% of parathyroid hyperplasia (Hsi, Zukerberg, Yang, & Arnold, 1996; Vasef, Brynes, Sturm, Bromley, & Robinson, 1999; Haven et al., 2004). The overexpression of cyclin D1 is associated with PC cell proliferation and a Ki‐67 index of ≥5% (Haven et al., 2004). Overexpression of *CCND1* gene may be associated with a 2–3 copy number gain of *CCND1*, which has been found to occur in five out of seven (∼70%) PCs (Figure 3), in contrast to the reported copy number gain of *CCND1* in only three out of 14 (∼20%) PAs (Zhao et al., 2014). The increased *CCND1* copy number in the PCs was associated with higher *CCND1* mRNA levels and protein expression. However, the mechanisms linking *CCND1* and PC tumorigenesis remain unknown. One hypothesis is that the potent inhibition of *CCND1* expression by *CDC73* may be lost after “two hits” on the *CDC73* gene, which may then trigger *CCDN1* disinhibition and tumorigenesis (Woodard et al., 2005).

### 4.6 EZH2

The enhancer of zeste 2 polycomb repressive complex 2 subunit (*EZH2*) gene is located on chromosome 7q36.1, and encodes a 746‐amino acid histone methyltransferase enzyme that directly controls gene methylation and transcriptional repression (Vire et al., 2006). *EZH2* mutations are rarely found in parathyroid tumors (Cromer et al., 2012; Sanpaolo et al., 2016). However, *EZH2* copy number gain (four gene copies) has been reported to occur in ∼60% of PCs (Figure 3), ∼30% of PAs, and 50% of parathyroid hyperplasia (Svedlund et al., 2014). Furthermore, PC samples without gene copy number gain showed increased levels of *EZH2* mRNA (Figure 3), suggesting the involvement of other indirect mechanisms (Svedlund et al., 2014). EZH2 may directly interact with β‐catenin inducing nuclear accumulation and activation of Wnt/β‐catenin signaling. EZH2 may also epigenetically repress Wnt antagonists like axis inhibition protein 2 (AXIN‐2), naked cuticle homolog 1 (NKD1), protein phosphatase 2 regulatory subunit B (PPP2R2B), prickle planar cell polarity protein 1 (PRICKLE1), and secreted frizzled related protein 5 (SFRP5), resulting in an increased activation of β‐catenin and increased expression of its target gene *CCND1* (Bjorklund, Akerstrom, & Westin, 2007; Li et al., 2009; Cheng et al., 2011). EZH2 represses, through histone modification H3K27me2/3, the tumor suppressor gene hypermethylated in cancer 1 (*HIC1*), which is involved in controlling growth of parathyroid cells and is reported to be decreased in PCs and PAs (Svedlund et al., 2012).

### 4.7 APC

Adenomatous polyposis coli (*APC*) is a tumor suppressor gene located on chromosome 5q22.2, that encodes a 2,843‐amino acid protein, which inhibits canonical Wnt signaling by controlling β‐catenin ubiquitination and proteolysis. Loss of *APC* expression has been reported in PCs, although *APC* mutations and copy number changes have not been observed, thereby suggesting that *APC* may be involved in epigenetic mechanisms (Figure 3) (Juhlin et al., 2010; Svedlund et al., 2010; Andreasson et al., 2012; Newey et al., 2012; Yu et al., 2015). Thus, APC expression is reported to be lost in 75% of PCs (Figure 3), but maintained in 100% of PAs (Juhlin et al., 2009). Quantitative real time PCR (qRT‐PCR) and Western‐blot analysis has also revealed that *APC* mRNA is either undetectable or very low, and that APC protein expression is undetectable in PCs (Svedlund et al., 2010). These alterations in APC expression in PCs may involve hypermethylation of the *APC* promoter 1A, and indeed methylation levels of *APC* promoter 1A CpGs were found to be significantly higher in PCs (>85%) than normal parathyroids (>15%); this was associated with decreased *APC* expression and accumulation of active nonphosphorylated β‐catenin (Svedlund et al., 2010). Moreover, treatment of PC cultured cells with the DNA methylation inhibitor 5‐aza‐2′‐deoxycytidine (decitabine) resulted in re‐expression of *APC* mRNA, APC protein, and reduced cell viability, thereby suggesting that decitabine could be an additional option in the treatment of patients with recurrent or metastatic PC (Svedlund et al., 2010).

### 4.8 GSK3B

Glycogen synthase kinase 3 beta (GSK3B) protein expression has been reported to be lost in <35% of PCs and ∼5% of PAs (Juhlin et al., 2009). The *GSK3B* gene, located on chromosome 3q13.33, encodes a 420‐amino acid enzyme regulating glycogen synthesis, Wnt, and PI3‐kinase/AKT signaling pathways. However, loss of GSK3B expression was not associated with any increase of β‐catenin or cyclin D1 expression, thereby suggesting that GSK3B may act through a pathway different to the classical Wnt/β‐catenin pathway in the etiology of PC (Juhlin et al., 2009). This would be consistent with results from several studies that have reported that abnormal nuclear expression of β‐catenin is not a characteristic of PC (Semba, Kusumi, Moriya, & Sasano, 2000; Juhlin et al., 2009; Cetani et al., 2010).

### 4.9 PRUNE2

Prune homolog 2 (*PRUNE2*) germline and somatic mutations, comprising three missense mutations (one germline mutation in a PC without *CDC73* mutations; two somatic mutations in two PCs without *CDC73* mutations) and two nonsense somatic mutations (c.1609G > T, p.Glu537X and c.1420G > T, p.Glu474X in a single PC with a *CDC73* mutation) have been reported to occur in four of 22 (∼20%) of PCs, but not PAs (Figure 3) (Yu et al., 2015). The *PRUNE2* gene, located on chromosome 9q21.2, encodes a 3,088‐amino acid protein that regulates cell differentiation and survival by suppression of Ras homolog family member A (RhoA) activity. *PRUNE2* has been reported to function as a tumor suppressor gene in prostate cancer, where prostate cancer antigen three (*PCA3*) regulates levels of PRUNE2 through formation of a *PRUNE2/PCA3* double‐stranded RNA (Salameh et al., 2015).

### 4.10 Epigenetic mechanisms of parathyroid carcinoma

Epigenetic mechanisms, which may involve histone methylation modifications and DNA methylation, have been reported to occur in PCs (Figure 3), and these include overexpression of *EZH2* and underexpression of *HIC1*, *APC*, and *GSK3B* as discussed above. Thus, the histone methyltransferases such as: EZH2, a H3‐lysine‐27‐methyltransferase enzyme; retinoblastoma protein‐interacting zinc finger gene 1 (RIZ1/PRDM2), a H3‐lysine‐9‐methyltransferase enzyme; and mixed lineage leukemia 2 (MLL2/KMT2D), a H3‐lysine‐4‐methyltransferase enzyme, have been reported to be involved in the pathogenesis of PC (Carling, Du, Fang, Correa, & Huang, 2003; Starker et al., 2011). Moreover, a somatic *MLL2/KMT2D* missense mutation (c.2522G > T, p.Cys841Phe) was reported in a PC, although in silico analysis has predicted that this is a likely tolerated/benign variant (Kasaian et al., 2013). Interestingly, menin and parafibromin, which are found to be mutated in such PCs, also interact with the histone methyltransferase SUV39H1 and function as transcription repressors by inducing H3K9 methylation (Rea et al., 2000; Yang et al., 2013). In addition, PCs have been reported to have promoter hypermethylation of: the Ras association domain family protein 1A (*RASSF1A*) gene, which encodes a Ras‐binding protein that down‐regulates cyclin D1 expression; and the secreted frizzled‐related protein 1 (*SFRP1*) gene, which was associated with epigenetic silencing and deregulated activation of the Wnt‐pathway (Figure 3). Hypermethylation of the promoters of the cyclin‐dependent kinase inhibitor 2A (*CDKN2A*), *CDKN2B*, Wilms tumor 1 (*WT1)*, *SFRP1*, *SFRP2*, *SFRP4*, and *RIZ1/PRDM2* genes, with reduced expression of the respective genes, have also have been reported in PCs, and expression of 5‐hydroxymethylcytosine (5hmC), an intermediate in DNA demethylation, was reported to be lower in PCs than in PAs (Starker et al., 2011; Sulaiman et al., 2013; Barazeghi et al., 2016). These results indicate that epigenetic mechanisms are likely involved in development of PC.

### 4.11 Role of microRNAs in parathyroid carcinoma

MicroRNAs (miRNAs) are small, 19–25 nucleotides long, non‐coding RNAs, that function as negative regulators of gene expression by decreasing translation or increasing degradation of the target mRNA. Studies of PCs have reported a global downregulation of approximately 60% miRNAs, when compared with normal parathyroid glands (Figure 3) (Corbetta et al., 2010; Rahbari et al., 2011). Downregulated miRNAs include miR‐26b, miR‐30b, miR‐126‐5p, miR‐296, and miR‐139 (Corbetta et al., 2010; Rahbari et al., 2011). Upregulated miRNAs include miR‐222, miR‐372, miR‐503, and miR‐517c (Corbetta et al., 2010; Vaira et al., 2012). These findings suggest that miRNA expression may contribute to development of PCs, and their role(s) remain to be elucidated.

### 5 GENETIC TESTING IN CLINICAL PRACTICE FOR PATIENTS WITH SUSPECTED PARATHYROID CARCINOMA

Distinguishing between PC (malignant disease) and PA (benign disease) on the basis of clinical and histological features is difficult and frequently not possible. This is because there is considerable overlap in the clinical features, including elevations of plasma calcium and PTH concentrations, and alkaline phosphatase activity, between patients with PC and PA (Wang & Gaz, 1985; Silverberg et al., 1990; Wynne et al., 1992; Chen et al., 2003). Moreover, histological examination may also not be able to reliably distinguish between PC, APA (an intermediate category between PC and PA), and PA (Bondeson, et al., 2004; DeLellis, 2011; Chan, 2013; Kumari et al., 2016). For example, it has been reported that use of pathological criteria has been associated with ≥50% of PCs, which actually behaved in a malignant manner being initially considered to be benign, and <15% of PCs were successfully diagnosed prospectively (Gill, 2014). However, *CDC73* mutational analysis and parafibromin immunostaining has been reported to be more reliable, with: *CDC73* mutations being identified in >75% of PCs but in <1% of PAs (Shattuck et al., 2003b; Krebs et al., 2005; Gill, 2014); and loss of nuclear parafibromin immunostaining occurring in >95% of PCs, but in <1% of PAs (Tan et al., 2004; Gill et al., 2006; Meyer‐Rochow et al., 2007). Moreover, even in the absence of a family history of PC or PAs, >30% of patients with PCs have a *CDC73* germline mutation, thereby indicating they had an unrecognized HPT‐JT syndrome or FIHP (Shattuck et al., 2003b; Cetani et al., 2004; Gill, 2014). These observations indicate that parafibromin immunostaining is useful for diagnosis of PC, and that genetic testing for germline *CDC73* mutations has an important role in the management of patients with proven or suspected PC, including APA, as these patients and their relatives are at risk of tumors associated with HPT‐JT. Identification of somatic mutations in some cancers is useful for targeting therapies, for example, epithelial growth factor receptor (EGFR) mutations for non‐small cell lung carcinoma (Paez et al., 2004; Gazdar, 2009); proto‐oncogene tyrosine‐protein kinase (KIT) mutations for chronic myeloid leukemia, gastrointestinal stromal tumors and melanoma (Heinrich, Blanke, Druker, & Corless, 2002; Willmore‐Payne, Holden, Tripp, & Layfield, 2005); or B‐Raf proto‐oncogene, serine/threonine kinase (*BRAF*) for melanoma and papillary thyroid cancer (Davies et al., 2002; Kimura et al., 2003). However, targeted therapies are not available for PC and at present genetic testing for somatic *CDC73* mutations using parathyroid tumor DNA may not be clinically useful for establishing the diagnosis or staging, especially as such tumors may contain multiple mutations. For example, whole exome sequence analysis of PCs and PAs have reported that the number of somatic mutations in these tumors vary between 3–176 and 2–110, respectively, and that <50% of these tumors may have *MEN1* mutations (Cromer et al., 2012; Newey et al., 2012; Yu et al., 2015). Moreover, our analysis, that has compared the frequency of somatic *CDC73* mutations in the catalogue of somatic mutations in cancer (COSMIC) database with the frequency of germline *CDC73* mutations in the exome aggregation consortium (ExAC) database, has revealed that there are ∼65‐fold more somatic non‐synonymous mutations than germline *CDC73* mutations. This increased frequency of somatic *CDC73* mutations is similar to that occurring for the neurofibromin 1 (*NF1*) gene, another tumor suppressor, in which somatic non‐synonymous mutations were ∼80‐fold more frequent than germline mutations. Recent studies have raised doubts about the pathogenicity of such mutations (Check Hayden, 2016; Lek, et al., 2016; Minikel, et al., 2016; Walsh et al., 2017), and while the clinical significance of such somatic mutations remains unknown, it would seem prudent at present, to reserve their investigation for research purposes only.

Genetic testing for germline *CDC73* mutations may be helpful in clinical practice in several ways including: (1) confirmation of the high risk for developing PC and associated syndromic and hereditary forms of PC, so that appropriate screening for associated tumors (e.g., PC, uterine tumors, renal tumors) can be undertaken; (2) implementation of appropriate treatment (e.g., early parathyroidectomy for patients with HPT‐JT because of increased risk of PC); (3) identification of family members who may be asymptomatic but harbor the mutation and therefore require screening for tumor detection and early treatment; and (4) identification of the 50% of family members who do not harbor the familial germline mutation and can therefore be relieved of the anxiety burden of developing tumors, while reducing the cost to the individuals and their children, and also to the health services in not having to undertake unnecessary biochemical and radiological investigations (Newey & Thakker, 2011; Thakker et al., 2012; Eastell et al., 2014; Thakker, 2016).

A genetic testing approach in a patient with a proven or suspected PC (Figure 1) could be as follows. The indications for undertaking such genetic testing in a patient are occurrence of: a proven or suspected sporadic PC or APA; a PA in association with an ossifying fibroma, early‐onset uterine tumor, renal cysts or tumor, or endocrine tumor (e.g., pancreatic neuroendocrine or pituitary tumor); PA or pHPT occurring <35 years of age; recurrent pHPT, multiglandular parathyroid disease or hyperplasia or FIHP (Figure 1 and Table 7). A detailed family history for the occurrence of hypercalcemia, pHPT, MEN1, MEN2, HPT‐JT, FIHP, and FHH should be obtained as the presence of these disorders in a relative will help to guide decisions for appropriate germline mutational analysis of the *MEN1*, *RET*, *CDC73*, or *CASR* genes, with the results of the tests further guiding clinical management and treatments (Figure 1). In the absence of a family history, the diagnosis of sporadic PC should be considered, and germline mutational analysis of *CDC73* undertaken as >30% of such patients will have a germline *CDC73* mutation, and will therefore be at high risk of developing HPT‐JT‐associated tumors (Figure 1) (Thakker et al., 2012; Eastell et al., 2014; Wells et al., 2015; Thakker, 2016). The first degree relatives of these patients, even if asymptomatic, should also be offered tests for germline *CDC73* mutations as these will help to identify if they have inherited the *CDC73* mutation and are therefore at high risk of developing HPT‐JT‐associated tumors (Figure 1), or not inherited the *CDC73* mutation in which case they can be reassured and have the burden of anxiety of developing PC and HPT‐JT‐associated tumors removed. Patients with sporadic PC who do not have *CDC73* mutations, are likely to have another etiology for their disease and should be offered the opportunity of participating in research studies to elucidate the genetic abnormalities causing this rare disorder (Thakker et al., 2012; Eastell et al., 2014; Wells et al., 2015; Thakker, 2016). Thus, identification of germline mutations would be helpful in the clinical management of PC patients and their families.

**Table 7:** Indications for *CDC73* mutational analysis

Sporadic PC
APA
Parathyroid tumor plus ossifying fibroma
Sporadic ossifying fibroma of the jaw
FIHP (*MEN1* and *CASR* mutations excluded)
PA or pHPT < 35 years (*MEN1* mutation excluded)
Recurrent pHPT (*MEN1* mutation excluded)
Multiglandular PA/hyperplasia (*MEN1* mutation excluded)
PA plus one or more of:
Early‐onset uterine lesion
Renal cysts/tumor
Pancreatic tumor
Thyroid tumor

PC, parathyroid carcinoma; APA, atypical parathyroid adenoma; FIHP, familial isolated primary hyperparathyroidism; *MEN1*, multiple endocrine neoplasia type 1; *CASR*, calcium sensing receptor; PA, parathyroid adenoma; pHPT, primary hyperparathyroidism.

Modified from Newey et al. (2010).

## 6 CONCLUSIONS

PC is a rare endocrine cancer, presenting typically with symptoms of hypercalcemia and predisposition to recurrence and metastasis. A definitive diagnosis of PC, which is usually based on histological analysis, is often only made retrospectively. Improvements in predicting the predisposition to PC and in diagnosis of PC are required, to facilitate improvements in patient care. To this end, molecular genetic studies have helped in identifying the underlying causes of PC and a genetic approach (Figure 1) can be helpful for the management of patients. Molecular genetic studies have revealed *CDC73* mutations to be major driver mutations in the etiology of PCs and defining and implementing clinical indications (Table 7) for *CDC73* mutation analysis will aid in future management and counseling of patients at risk from PC and PC‐associated syndromes such as HPT‐JT. The genetic etiology causing PC involves other genes, which include *MEN1*, *RET*, and *PRUNE2*, as well as epigenetic mechanisms, alterations in miRNA expression and potentially as yet unidentified genes. PC is a rare neoplasm, and it is therefore essential that collaborative efforts that pool scarce tumor material and increase sample size are pursued to facilitate the identification of the genetic etiology of PC by next generation sequencing methodologies. These approaches are likely to yield important insights into the causative mechanisms for PC and to improved methods at detecting and diagnosing PCs, whose translation into the clinic are likely to lead to improved treatments and outcomes for patients.
